# Metabolic differentiation of brushtail possum populations resistant and susceptible to plant toxins revealed via differential gene expression

**DOI:** 10.1007/s00360-024-01591-z

**Published:** 2024-11-04

**Authors:** David Carmelet-Rescan, Mary Morgan-Richards, Steven A. Trewick

**Affiliations:** Wildlife and Ecology, School of Natural Sciences, Massey University, Private Bag 11-222, Palmerston North, New Zealand

**Keywords:** 1080, Brushtail possums, Differential expression, Candidate genes, RNA-Seq, Sodium fluoroacetate, Toxin tolerance

## Abstract

The Australian brushtail possum (*Trichosurus vulpecula*) is adapted to a wide range of food plants across its range and is exposed to numerous physiological challenges. Populations that are resistant to the plant toxin sodium fluoroacetate are of particular interest as this compound has been used since the 1940s for vertebrate pest management around the world. Candidate gene identification is an important first step in understanding how spatial populations have responded to local selection resulting in local physiological divergence. We employ differential gene expression of liver samples from wild-caught brushtail possums from toxin-resistant and toxin-susceptible populations to identify candidate genes that might be involved in metabolic pathways associated with toxin-resistance. This allowed us to identify genetic pathways involved in resistance to the plant toxin sodium fluoroacetate in Western Australian possums but not those originally from south eastern Australia. We identified differentially expressed genes in the liver that are associated with cell signalling, encapsulating structure, cell mobility, and tricarboxylic acid cycle. The gene expression differences detected indicate which metabolic pathways are most likely to be associated with sodium fluoroacetate resistance in these marsupials and we provide a comprehensive list of candidate genes and pathways to focus on for future studies.

**Supplementary Information:**

The online version contains supplementary material available at 10.1007/s00360-024-01591-z.

## Introduction

In natural environments, plants are engaged in reciprocal selective interactions with the animals that eat them (Ehrlich and Raven 1964). This coevolutionary arms race (Dawkins and Krebs 1979; Endara et al. 2017) involves many types of weapons with plant primary and secondary metabolites playing a major role in the armoury. The production of plant secondary metabolites that are toxic to herbivores and so protect plant foliage are implicated in the reciprocal evolution of toxin-resistance, which involves the development of mechanisms by organisms to counteract the harmful effects of toxins produced by their counterparts. Enhanced resistance to particular toxins has been extensively studied in numerous ecological contexts, and understanding the mechanisms of this resistance is essential for elucidating the complex arms race between species and the coevolutionary processes underlying it (e.g. de Castro et al. 2018). These mechanisms allow animals to either bind and inactivate toxins, render target molecules refractory to toxins, or exploit toxin functions to alter their physiological effects. Where herbivores exist that vary in their sensitivity to the same toxin, candidate gene identification via differential gene expression is an important first step in understanding the mechanisms animals have evolved to prevent plant toxins killing them.

Advances in high-throughput sequencing technologies have facilitated the investigation of gene expression differences among populations and species of non-model organisms and allowed identification of candidate genes associated with numerous important physiological adaptations (Stanford et al. 2020). Recently, the tools and platforms available for RNA-seq research have rapidly expanded, reducing costs but adding potential confounding factors into analyses. Differential gene expression studies have provided valuable insights into the genetic mechanisms underlying phenotypic diversity, as well as the evolutionary processes that shaped it (Costa-Silva et al. 2017; Todd et al. 2016; Zhang et al. 2014). In this study, we explore differential gene expression of two populations of brushtail possum; the natural Western Australian population and the population introduced to New Zealand, representing two subspecies of *Trichosurus vulpecula* (How and Kerle 1995; Kerle et al. 1991). Specifically, we focus on expressed genes in the liver of adult brushtail possums that might be involved in resistance to plant toxins, an important trait that distinguishes the Western Australian subspecies (*T. v. hypoleucus*) from the subspecies in Tasman (*T. v. fuliginosis*) and east Australia (*T. v. vulpecula*). These latter subspecies are now present as a hybrid swarm in New Zealand (Bond et al., 2023; Pattabiraman et al. 2021).

In Australia, the brushtail possum (*Trichosurus vulpecula*) has a wide geographic range from the southwest to the northeast of the country. The different lineages within this species are considered subspecies despite their morphological similarities (Kerle et al. 1991). The main delimiting factors are the geographical distance separating populations and fur colour patterning, but genetic data (Campbell et al. 2021; Pattabiraman et al. 2021; Taylor et al. 2004) and a time-calibrated phylogeny (Carmelet-Rescan et al. 2022) indicate lineage differentiation consistent with the subspecies’ dating to the Pliocene (between 3.5 and 2.5 Mya). Forage on local flora exposes possums to different plant defence regimes across their geographic range, driving local adaption. In Western Australia, *T. v. hypoleucus* brushtail possums consume several plants that are rich in plant defence chemicals (e.g. *Erythophleum*,* Acacia*,* Eucalyptus* and *Gastrolobium*). *Gastrolobium* species, for example, contain high concentrations of sodium fluoroacetate (Abubakari et al. 2021) and here brushtail possums have an LD50 160 times higher than the populations in east Australia (*T. v. fuliginosis* and *T. v. vulpecula*) (Leong et al. 2017; Twigg et al. 1996). Sodium monofluoroacetate (Compound 1080) was first used to kill rats in the USA in the 1940s and is now widely used for the control of mammal pest species (Cooper et al. 2007; Sherley 2004). The emergence of physiological adaptations within brushtail possum populations specific to regional flora indicates intense, local, natural selection resulting in genomic divergence of spatial populations (Mead et al. 1985; Oliver and King 1979). Geographic isolation likely helped maintain resistance in *T. v. hypoleucus* in contrast to populations elsewhere (Twigg and King 1991) including the toxin susceptible possums (*T. v. fulginosis/vulpecula*) introduced to New Zealand (How and Kerle 1995; Kerle et al. 1991).

In New Zealand invasive possums are a vector for bovine tuberculosis (TB) which is costly for the agricultural industry. Invasive possums are also highly destructive of native fauna and flora (Byrom et al. 2015; Nugent et al. 2015) and are placed on the target list for eradication in New Zealand (Tompkins 2018). The application in New Zealand of synthetic 1080 (McIlroy 1983; Ross et al. 1999) to a million hectares each year results in repeated high local kill, as aerial drops can reach more than 80% efficiency (Nugent et al. 2012). However, this is followed by rapid population recovery (Cowan 1992, 2016; Gupta 2015; Ross et al. 1999) requiring repeated poisoning episodes. This management practice favours the evolution of toxin resistance in New Zealand, just as occurred in wild Western Australian possums and within experimental systems (Brown and Payne 1988). The evolution of elevated resistance to 1080 in New Zealand possums would have major implications for agriculture and conservation, which is why determining the underlying basis of toxin resistance is crucial for the future of possum management in New Zealand.

Understanding the genetic mechanisms behind this toxin resistance is essential for maintaining effective control measures. There are many possible genetic paths that might lead to herbivore resistance to plant toxins (Bomblies and Peichel 2022), but several are likely to involve a change in the level of expression of existing genes (Adamczyk et al. 2001; Jenkins et al. 2015). Differential expression analysis using messenger RNA Seq data is a relatively tractable route for comparative analysis of gene expression among locally adapted populations and species (Boaventura et al. 2021; Cortes et al. 2020; Porcelli et al. 2016). When comparing expression data from different possum lineages we expected to find many differentially expressed genes associated with a wide range of cellular pathways due to the phylogenetic distance between the subspecies (Carmelet-Rescan et al. 2022). However, we predicted that genes that are associated with metabolic pathways involved in detoxification mechanisms such as acetate will be differentially expressed in the liver. The toxin sodium fluoroacetate (1080) interferes with the biochemical pathway that generates energy (the tricarboxylic acid cycle) and we therefore expected to detect differential expression of genes involved in carbon metabolism. We generated RNASeq data from fresh liver samples of brushtail possums from 1080-resistant *T. v. hypoleucus* (Western Australia) and 1080-susceptible *T. v. fulginosis/vulpecula* (New Zealand) populations and performed differential gene expression analysis to identify candidate genes. We then investigated the pathways associated with the genes showing evidence of differential expression to identify potential associations with resistance to toxins and/or sodium fluoroacetate metabolism.

## Material and method

Our analysis includes a comparison of New Zealand adult and juvenile gene expression from published material (Bond et al. 2023) that allows removal of genes associated with development from downstream analyses. The legal protection of brushtail possums in Western Australia limited the number of individuals available for comparison with a larger sample from New Zealand where the species is an invasive pest. To ensure this uneven sampling does not have a significant negative effect on downstream analyses we undertook a power analysis to determine the influence of small and imbalanced sample size in candidate gene identification.

### Sample collection

Fresh liver samples were collected by vets from three wild adult brushtail possums in Perth, Western Australia that were euthanised because of mortal injuries resulting from being struck by motor vehicles. These individuals represent the toxin-resistant (hereafter ‘resistant’) subspecies (*T. v. hypoleucus*). The livers of nine wild-caught adult brushtail possums were sampled across New Zealand (Turitea, Manawatū, North Island *n* = 3; Manaroa, Marlborough, South Island, *n* = 2; Stewart Island *n* = 4) from individuals killed during local pest control. These New Zealand samples represent toxin-susceptible (hereafter ‘susceptible’) possums brought from southeast Australian and Tasmanian subspecies (*T. v. fuliginosus* and *T. v. vulpecula*) in the nineteenth century (Campbell et al. 2021; Pattabiraman et al. 2021; Pracy 1974). The liver tissues were immediately immersed in ample RNALater (Invitrogen) preservation liquid and stored at -20 °C until RNA extraction (Table 1).

### RNA extraction and sequencing

The instability of messenger RNA makes it challenging to transport samples between New Zealand and Western Australia and COVID restrictions at the time of this research further limited options. Therefore a number of approaches were applied during data generation.

Total RNA was extracted from the Western Australian possum samples using the Qiagen RNeasy Mini Kit automated on a QiaCube according to the manufacturer’s instructions including DNase digestion at AGRF Ltd (Melbourne, Victoria, Australia). RNA was extracted from the nine fresh New Zealand samples at Massey University, New Zealand using the Nucleopsin RNA (Macherey-Nagel) after grinding in liquid nitrogen, according to the manufacturer’s protocol. Extractions were followed by DNA removal using DNase prior to library preparation. The concentration of DNase-treated RNA was determined using the Qubit RNA BR Assay Kit with the Qubit Fluorometer (Invitrogen). Quantity and quality were assessed using PerkinElmer LabChip^®^ GX Touch HT.

Library preparations were performed according to the sequencing platform and the sample quality. Poly(A) enriched libraries and rRNA removal were preferred for higher quality samples. Two of the Western Australian samples were considered suboptimal for poly-A capture and were instead sequenced using whole-transcriptome sequencing on a NovaSeq RNA Sequencing platform at AGRF Melbourne generating 150 bp PE reads. Samples from Stewart Island (New Zealand) were also sequenced using a NovaSeq platform via Custom Science Ltd NGS services (Auckland, New Zealand) generating 150 bp PE reads. Other samples were sequenced on the DNBSeq platform from BGI Tech Solutions Ltd (Tai Po, Hong Kong) producing 100 bp PE reads.

Prior to mapping, the quality of the sequence reads was assessed using FastQC (Andrews 2010) and adapter sequence and quality-based trimming was performed using Trimmomatic (Bolger et al. 2014) removing reads ≤ 20 bp in length and reads with a quality score of ≤ 20. Sequences were then aligned to the brushtail possum (*Trichosurus vulpecula*) genome (Genebank: mTriVul1.pri - GCA_011100635.1, Annotation Release 100) using HISAT2 (Kim et al. 2019), a spliced read aligner, with default parameters. Samtools (Li et al. 2009) was used to sort, format and output.bam alignment files, and the Picards tools (Institute 2019) were implemented to collect alignment quality metrics. RNA library preparation of most samples (AZENTA (Custom Science), Otago Genomic, and BGI Genomics) included oligo_dT treatment, a technique to minimise ribosomal RNA in samples. However this was not done for two samples (WA1, WA2) leading to larger representation of rRNA sequences among the resulting data, and so the rRNA gene sequences were removed in silico before analyses.

Publicly available RNASeq data generated prior to this study from liver samples representing a single possum population in Otago (New Zealand) were included in preliminary analyses (GenBank Bioproject: PRJNA904814, (Bond et al., 2023). This comprised 23 individuals with 14 adults and 9 juveniles.

### Differential expression analysis

The number of RNA reads mapped to each gene was reported using featureCounts software (Liao et al. 2014), providing a table of the number of mapped reads for each gene that form the base data for differential expression analysis. Feature-specific quantile normalisation was applied to the read count variance within each category to eliminate any platform-based bias using the R package “FSQN” v 0.0.1 (Franks et al. 2018). This eliminates distribution-based differences resulting from the use of different gene expression profiling platforms. Using more than one pipeline to compute differential expression analyses potentially broadens the resulting set of candidate genes and helps identify concordant signal for particular genes. We used three distinct approaches to identify differentially expressed genes: DESeq2 (Love et al. 2014) which fits the data to a negative binomial generalized model; Limma using linear modelling (Law et al. 2014; Phipson et al. 2016; Ritchie et al. 2015); and weighted gene co-expression network analysis (WGCNA) (Langfelder and Horvath 2008). DESeq2 and Limma are recognised as having a low rate of false positives (Seyednasrollah et al. 2013) and are expected to display a clear overlap. WGCNA is a very different approach that compares data with co-expression modules that each comprise many genes, and so could reveal associations not detected by other algorithms. In combination these approaches could provide more nuanced results (Bao et al. 2021; Farhadian et al. 2021). Here we present results from the DESeq2 pipeline, with details of the Limma and WGCNA analyses in supplementary material. A flowchart detailing the full methods of the study is represented in Supplementary Fig. 1.

### Differential gene expression analysis based on the negative binomial distribution (DESeq2)

The DESeq (Love et al. 2014) function estimates the size factor (length of the gene) and dispersion before fitting the data to a negative binomial generalized model and computing the Wald statistic for significance testing. Previous studies showed that parametric models are appropriate when replicates are few (Kim et al. 2019) and that DESeq2 responds to higher read depth by assigning smaller p-values to transcripts with small fold-change (Robles et al. 2012). Controlling the false discovery rate is an essential step of every differential expression study (Korthauer et al. 2019) and this is incorporated in the DESeq2 pipeline using Benjamini and Hochberg’s step-up procedure (Benjamini and Hochberg 1995; Love et al. 2014) that adjusts the p-value. Significantly differentially expressed genes (DEGs) were conservatively selected using FDR-adjusted p-values lower than 0.00001. A log fold change of expression level above 2 or below − 2 (corrected using normal shrinkage estimation) was used to improve stability and interpretability (Love et al. 2014), but results between those thresholds were also explored. Significant DEGs between adult resistant and susceptible possums were thus obtained. Principal component analysis, clustered heatmapping of the significant DEGs and volcano plots were performed for visualisation using the R package “DESeq2” v1.44.0 (Love et al. 2014) and EnhancedVolcano v1.22 (Kevin Blighe et al., 2024).

Significant DEGs associated with possum development were obtained using the population sample of liver RNA from sixteen adults and seven juveniles from Otago. This allowed identification of expression differences that result from the age of the individual and these were filtered and excluded from further analysis so that expression differences between subspecies were enriched while any effect of misclassified age class and unequal representation of rRNA sequences among samples was minimised.

A supporting analysis considering sample size was performed based on the comparison of gene expression in adult and juvenile possums (Full details are in Supplementary Material 2). Briefly, we repeated our differentiation expression analysis comparing adult and juveniles altering the number of randomly selected individuals in each of the two groups and compared the set of candidate genes identified in each re-sampling analysis with the set obtained when the full dataset was sampled. We repeated these analyses with two pipelines and four p-values to assess the effects of sample size and unbalanced sampling in the identification of candidate genes using differential gene expression. Results showed that even with a small number of individuals and potentially unbalanced sampling the data can yield reliable and informative results.

Since the *Trichosurus vulpecula* genome is not included in the database of GOs (Gene Ontologies) and KEGG (Kyoto Encyclopedia of Genes and Genomes) pathways we used the genome (*Pan troglodites*) that returned the highest proportion of common genes (> 83% of found genes) to identify putative GO terms and KEGG pathways associated with those genes. Gene Ontology enrichment analysis was also computed on the significantly differentially expressed genes using the “goseq” v1.56.0 and “clusterProfiler” v4.12.2 packages (Young et al. 2013; Yu et al. 2012) to determine the over-represented GO terms present in the list. The significance of enriched GO terms was determined using Wallenius non-central hypergeometric distribution (Wallenius 1963) of significantly differentially expressed genes p-values, including Benjamini & Hochberg FDR control (Benjamini and Hochberg 1995). Those GO terms and their parent relations were explored using REVIGO (Supek et al. 2011) and visualized as circle charts using the program CirGO (Kuznetsova et al. 2019). KEGG enrichment analysis used the enrichKEGG function of the “clusterProfiler” R package (Yu et al. 2012) that includes an FDR control. The significantly enriched pathways alongside the associated under-expressed and over-expressed genes were then plotted using the “enrichplot” v1.24.2 R package (Yu 2021).

### Differential expression analysis based on linear model (Limma)

The data were also analysed using an approach with a different normalization process implemented in the “Limma” v3.60.4 R package (Ritchie et al. 2015). The first step calculates the scaling factor for each gene from the count data according to the library size and then transforms the counts to normalized log2-counts per million. The next step fits multiple linear models and then computes associated statistics needed to select the significantly differentially expressed genes. The p-value cutoff for significance was set to 0.00001 and log2-fold over 2 and under − 2. GO enrichment analysis was performed on Limma DEGs in the same way as DESeq2 DEGs.

### Weighted correlation network analysis (WGCNA)

Using the weighted correlation network analysis and the “WGCNA” v1.72-5 R package (Langfelder and Horvath 2008), genes with more than 50% missing data were filtered out yielding 15,674 studied genes. Expression values were normalized using DESeq2 variance stabilisation. The function “hclust” from the package “fastcluster” v1.2.6 (Müllner 2013) applied to the expression results clusters samples and excludes those that deviate based on cluster height value (> 150). The construction of the co-expression network was realized using Pearson’s correlation and then the adjacency matrix using the function a_mn_ =|c_mn_|^b^ (*a*_*mn*_: adjacency between gene m and gene n, *c*_*mn*_: Pearson’s correlation, b: soft-power threshold). Soft-power threshold selection uses the lowest power for which the scale-free topology fit index reaches 0.80. Noise and spurious association effects were minimized by transforming the adjacency matrix into a Topological Overlap Matrix and then calculating the associated dissimilarity. Hierarchical clustering on the TOM-based dissimilarity is then performed to produce a hierarchical clustering dendrogram of genes. The dendrogram modules were identified using Dynamic Tree Cut and similar modules merged based on co-expression similarity of entire modules. Module association with the traits of interest (population) were quantified by performing principal components analysis of each module; the first components of each module are referred to as module eigengenes (MEs). Pearson correlation between the traits and the MEs is then calculated with associated P-values. Statistically significant modules (*P* < 0.01) were selected to inspect for significantly differentially expressed genes. Gene significance (GS) was computed as the correlation between each individual gene of a module with the biological trait (subspecies), and module membership (MM) as the correlation between the gene and the module expression profile. Significant genes were then determined using a threshold of MM > 0.8 and GS > 0.8 in the significant modules. Similarly, gene ontology enrichment analysis was performed on significant DEGs discovered by WGCNA using the same method as above.

To illustrate the results of the three different analyses proportional Venn diagrams were computed using the R package “eulerr” v7.0.2 (Larsson and Gustafsson 2018).

## Results

The times and places of sampling possums coupled with constraints on tissue preservation required RNA extraction and sequencing to be done in several different laboratories. There were more samples of susceptible than resistant possums and provenance of samples and the sequencing provider are correlated. The different sequencing technologies generated differing numbers of reads and reads of differing length. Initial analysis revealed a relatively high number of unmapped reads from the AGRF platform, and the Otago samples yielded lower sequence depth. Additionally all samples displayed more than 20% of reads mapping to unannotated segments of the reference genome (Supplementary Fig. 2). Reads mapping to non-annotated parts of the possum genome, and elevated number of rRNA sequences reflect technical issues including partial failure of the poly-A capture step and rRNA depletion steps during the library preparation process stage (Tellier and Murphy 2020). However, the samples concerned generated high numbers reads that balanced those issues and the overall quality of the reads was good among all samples (Table 1). Use of normalization techniques ensured that the despite sample variation we can be confident in our inferences of differential gene expression.

### Differential expression of juvenile and adult brushtail possums

Differential expression analysis was performed using mapped read count from the Otago population sample to compare 14 adults with 9 juveniles (Table 1) across 15,662 genes. After analysis using DESeq2, normalisation, shrinkage and FDR analysis, 475 genes were classified as showing significantly different transcriptional levels (more than a log2 fold difference of two with an FDR adjusted p-value less than 0.00001). Most of these genes were associated with the cell cycle and protein digestion (Supplementary Figs. 3 and 4). Among the 475 genes, 302 were up-regulated and 173 down-regulated in juveniles compared to adults, suggesting developmental changes in *Trichosurus vulpecula.* This was consistent with previous analyses that identified developmental change in expression levels of cytochrome P450-family (CYP) genes (Bond et al., 2023).

Our simulation study in which sample size was varied to determine whether we could identify the same set of candidate genes differentiating adults from juveniles revealed a clear relationship between the proportion of genes identified and sample size. However, having data from more individuals in one group was shown to resolve differential gene expression well. We confirmed that three individuals per population sample was better than two, but three in one group and 14 in another resulted in better DEG discovery. More than 50% of the candidate genes were found using *n* = 3 and *n* = 14, compared to only 31% of common significant DEGs with *n* = 3 and *n* = 3 (Details in Supplementary Material 2). This simulation indicated that the unbalanced sampling used in our search for candidate genes for toxin resistance will be informative.

### Differential expression patterns among brushtail possum common to three analysis pipelines

RNA-Seq analysis was performed on liver samples of adult brushtail possums (*Trichosurus vulpecula*) to explore gene expression differences between two major lineages of brushtail possums. Specifically, these comprised three resistant and 23 susceptible possums, representing two major lineages within this species (*T. v. hypoleucus*, and *T. v. fuliginosis*/*vulpecula*). A strict quality control analysis for each sample was conducted to confirm the quality of the reads, yielding more than 13 million reads of retained clean data per sample, representing a total of > 1.3 Gbps (Table 1).

**Table 1:** Samples of brushtail possum (*Trichosurus vulpecula*) used to study differential expression of genes in the liver. Details of the quantity and quality of the RNA sequences for each sample provided. Samples with codes OT are from Bond et al. 2023 Toxin resistant refers to ability to tolerate Sodium monofluoroacetate (1080)

Sample code	Origin	Maturity	Library Preparation	Sequencing platform	Read Size (PE)	Number of reads (Millions)	Base pairs (Gb)	Q20 bases (%)	TOxin-RESISTANT
OT_J_1	Otago, New Zealand	**JUVENILE**	Poly(A)-enriched, oligo_dT treatment	HiSeq 2000 (Illumina)	100 bp	16.97	1.71	92.35	**NO**
OT_J_2	Otago, New Zealand	**JUVENILE**	Poly(A)-enriched, oligo_dT treatment	HiSeq 2000 (Illumina)	100 bp	20.49	2.07	92.65	**NO**
OT_J_3	Otago, New Zealand	**JUVENILE**	Poly(A)-enriched, oligo_dT treatment	HiSeq 2000 (Illumina)	100 bp	16.07	1.62	90.77	**NO**
OT_J_4	Otago, New Zealand	**JUVENILE**	Poly(A)-enriched, oligo_dT treatment	HiSeq 2000 (Illumina)	100 bp	26.32	2.66	92.63	**NO**
OT_J_5	Otago, New Zealand	**JUVENILE**	Poly(A)-enriched, oligo_dT treatment	HiSeq 2000 (Illumina)	100 bp	23.22	2.34	91.88	**NO**
OT_J_6	Otago, New Zealand	**JUVENILE**	Poly(A)-enriched, oligo_dT treatment	HiSeq 2000 (Illumina)	100 bp	22.94	2.31	92.94	**NO**
OT_J_7	Otago, New Zealand	**JUVENILE**	Poly(A)-enriched, oligo_dT treatment	HiSeq 2000 (Illumina)	100 bp	29.98	2.77	88.76	**NO**
OT_J_8	Otago, New Zealand	**JUVENILE**	Poly(A)-enriched, oligo_dT treatment	HiSeq 2000 (Illumina)	100 bp	22.22	2.24	92.08	**NO**
OT_J_9	Otago, New Zealand	**JUVENILE**	Poly(A)-enriched, oligo_dT treatment	HiSeq 2000 (Illumina)	100 bp	33.00	3.33	92.30	**NO**
OT_1	Otago, New Zealand	ADULT	Poly(A)-enriched, oligo_dT treatment	HiSeq 2000 (Illumina)	100 bp	26.08	2.63	92.43	**NO**
OT_2	Otago, New Zealand	ADULT	Poly(A)-enriched, oligo_dT treatment	HiSeq 2000 (Illumina)	100 bp	26.98	2.72	92.00	**NO**
OT_3	Otago, New Zealand	ADULT	Poly(A)-enriched, oligo_dT treatment	HiSeq 2000 (Illumina)	100 bp	34.98	3.53	90.91	**NO**
OT_4	Otago, New Zealand	ADULT	Poly(A)-enriched, oligo_dT treatment	HiSeq 2000 (Illumina)	100 bp	25.61	2.58	92.00	**NO**
OT_5	Otago, New Zealand	ADULT	Poly(A)-enriched, oligo_dT treatment	HiSeq 2000 (Illumina)	100 bp	17.68	1.78	92.41	**NO**
OT_6	Otago, New Zealand	ADULT	Poly(A)-enriched, oligo_dT treatment	HiSeq 2000 (Illumina)	100 bp	24.10	2.43	92.38	**NO**
OT_7	Otago, New Zealand	ADULT	Poly(A)-enriched, oligo_dT treatment	HiSeq 2000 (Illumina)	100 bp	27.89	2.81	92.50	**NO**
OT_8	Otago, New Zealand	ADULT	Poly(A)-enriched, oligo_dT treatment	HiSeq 2000 (Illumina)	100 bp	17.65	1.78	92.30	**NO**
OT_9	Otago, New Zealand	ADULT	Poly(A)-enriched, oligo_dT treatment	HiSeq 2000 (Illumina)	100 bp	39.96	4.03	91.89	**NO**
OT_10	Otago, New Zealand	ADULT	Poly(A)-enriched, oligo_dT treatment	HiSeq 2000 (Illumina)	100 bp	27.26	2.75	92.12	**NO**
OT_11	Otago, New Zealand	ADULT	Poly(A)-enriched, oligo_dT treatment	HiSeq 2000 (Illumina)	100 bp	28.54	2.88	91.33	**NO**
OT_12	Otago, New Zealand	ADULT	Poly(A)-enriched, oligo_dT treatment	HiSeq 2000 (Illumina)	100 bp	13.02	1.31	91.51	**NO**
OT_13	Otago, New Zealand	ADULT	Poly(A)-enriched, oligo_dT treatment	HiSeq 2000 (Illumina)	100 bp	25.09	2.53	92.74	**NO**
OT_14	Otago, New Zealand	ADULT	Poly(A)-enriched, oligo_dT treatment	HiSeq 2000 (Illumina)	100 bp	24.76	2.50	93.32	**NO**
MA_1	Manaroa, New Zealand	ADULT	Poly(A)-enriched, oligo_dT treatment	DNBSeq platform	150 bp	46.04	4.59	87.94	**NO**
MA_2	Manaroa, New Zealand	ADULT	Poly(A)-enriched, oligo_dT treatment	DNBSeq platform	150 bp	40.90	4.01	82.71	**NO**
ST_1	Stewart Island, New Zealand	ADULT	Poly(A)-enriched, oligo_dT treatment	NovaSeq	150 bp	58.45	8.73	91.68	**NO**
ST_2	Stewart Island, New Zealand	ADULT	Poly(A)-enriched, oligo_dT treatment	NovaSeq	150 bp	34.62	5.15	91.58	**NO**
ST_3	Stewart Island, New Zealand	ADULT	Poly(A)-enriched, oligo_dT treatment	NovaSeq	150 bp	36.64	5.46	91.54	**NO**
ST_4	Stewart Island, New Zealand	ADULT	Poly(A)-enriched, oligo_dT treatment	NovaSeq	150 bp	45.79	6.84	91.29	**NO**
MN_1	Manawatu, New Zealand	ADULT	Poly(A)-enriched, oligo_dT treatment	DNBSeq platform	150 bp	26.49	2.64	91.56	**NO**
MN_2	Manawatu, New Zealand	ADULT	Poly(A)-enriched, oligo_dT treatment	DNBSeq platform	150 bp	25.29	2.47	94.35	**NO**
MN_3	Manawatu, New Zealand	ADULT	Poly(A)-enriched, oligo_dT treatment	DNBSeq platform	150 bp	24.90	2.48	90.16	**NO**
WA_1	**Western Australia**	ADULT	Whole Transcriptome	NovaSeq	150 bp	40.56	5.89	89.86	**YES**
WA_2	**Western Australia**	ADULT	Whole Transcriptome	NovaSeq	150 bp	59.22	8.59	89.84	**YES**
WA_3	**Western Australia**	ADULT	Poly(A)-enriched, oligo_dT treatment	DNBSeq platform	100 bp	14.78	1.47	92.59	**YES**

**Fig. 1:**
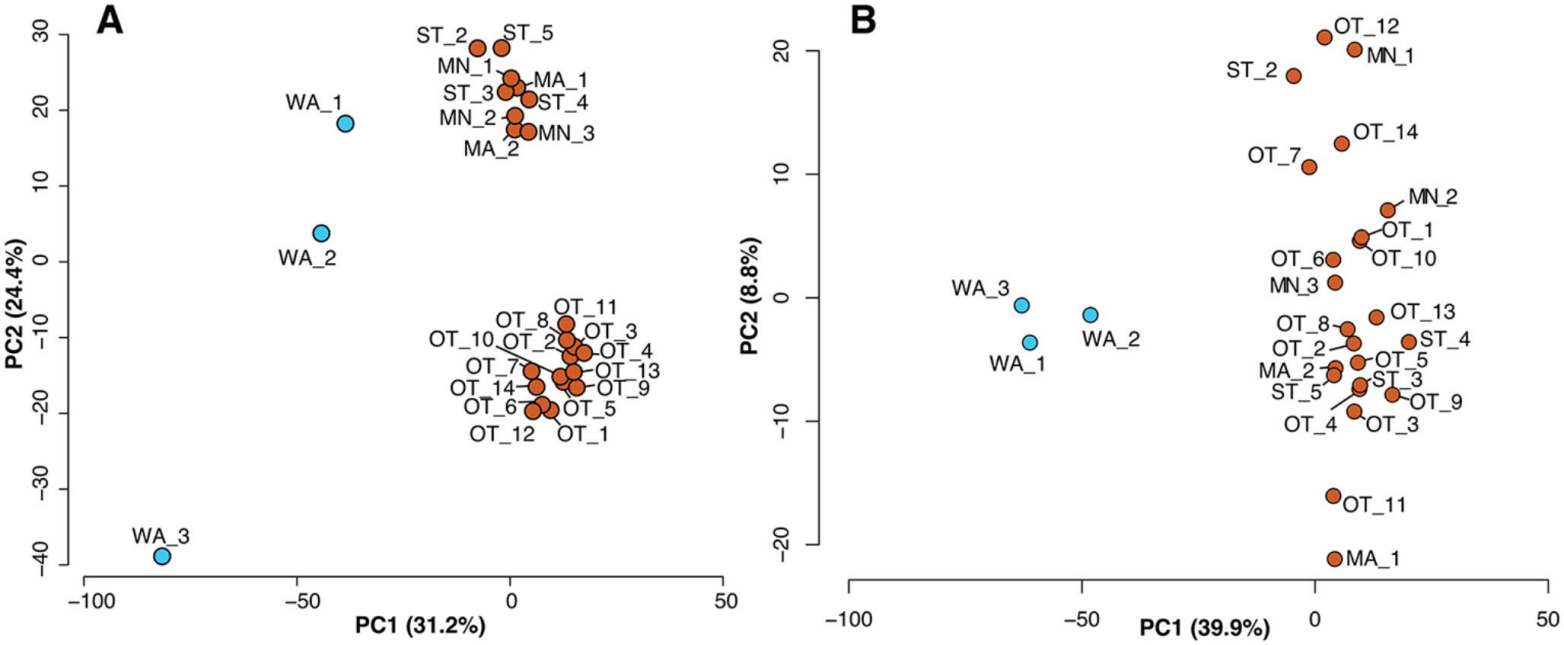
The distribution and separation of brushtail possum (*Trichosurus vulpecula*) liver samples based on their gene expression patterns, highlighting differences among samples. Principal Component Analysis plot showing the results of (**A**) differential gene expression analysis applied to the normalized count data, and (**B**) differential gene expression analysis applied to the normalized count data after feature-specific quantile normalisation (FSQN). Each dot represents an individual: resistant (blue) or susceptible (orange) to 1080-toxin and the eigenvalue proportion of axis indicated

Exploratory principal component analysis of the normalized counts from the differential gene expression analysis using DESeq2 (Love et al. 2014) clustered samples according to their sequencing platform (Fig. 1A). However, this clustering disappeared following feature-specific quantile normalisation (FSQN) and the principal differences in gene expression patterns were resolved between data from the three resistant and the 23 susceptible adult brushtail possums (PC1 = 39.9% of variation; Fig. 1B).

**Fig. 2:**
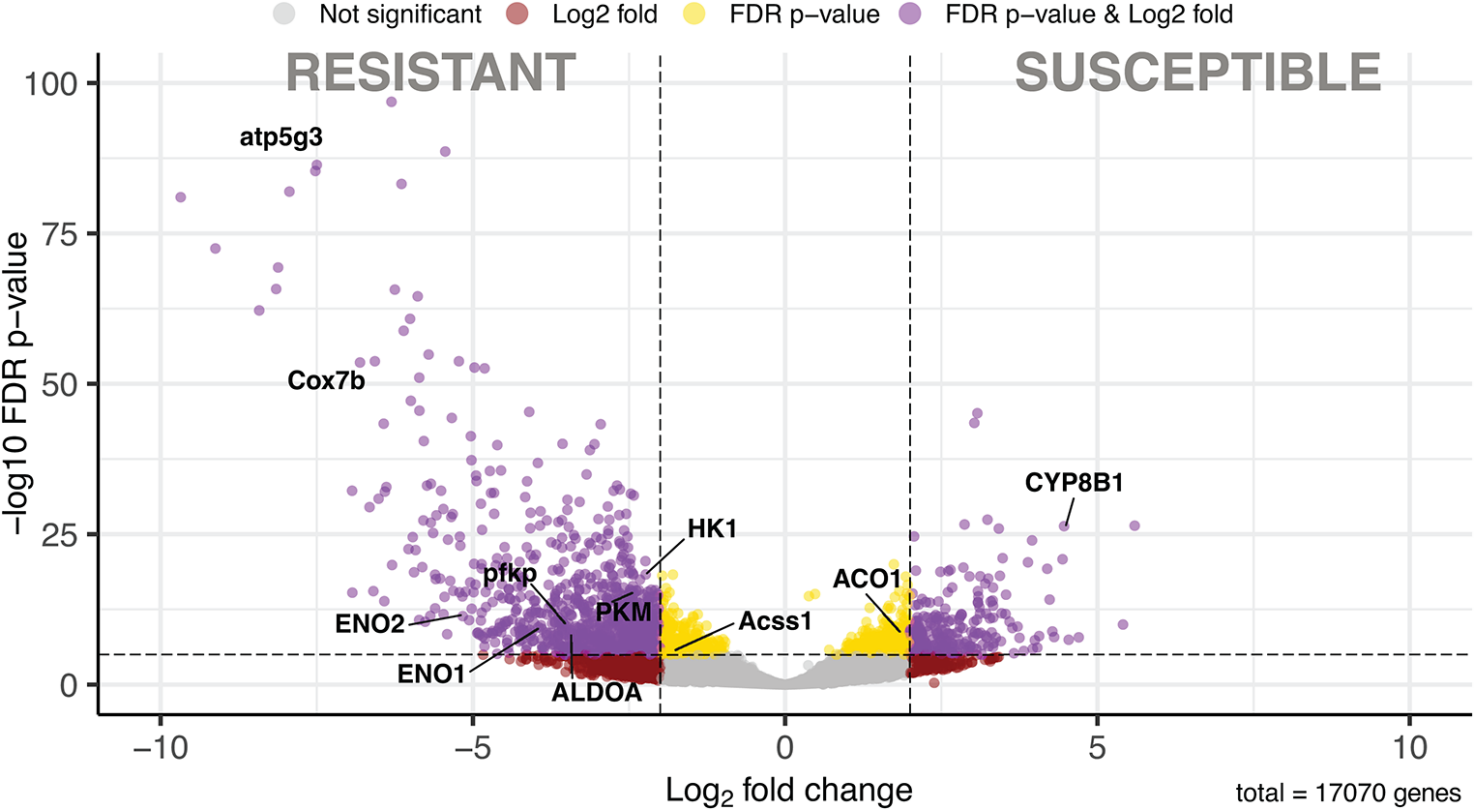
Volcano plot of differentially expressed genes (DEGs) in livers of resistant (*Trichosurus vulpecula. hypoleucus*) versus susceptible (*T. v. fuliginosis*/*vulpecula*) brushtail possum using the R packages “DESeq2”. The representations are as follows: x-axis, log2 fold change; y-axis, -log10 of the FDR adjusted p-value. The genes with p-values < 0.00001 are the yellow dots, and the genes with logFC ≥ 2 and logFC ≤ − 2 are the red dots; the significant DEGs satisfying both value thresholds are in purple and genes of interest discussed in the text are indicated with abreviations. Grey dots indicate the remaining genes present in the array that were not significantly differently expressed. Genes upregulated in resistant and susceptible are on the left and right respectively

Significantly differentially expressed genes were selected with a p-value threshold of 1e-05 and a log fold change of 2 (Fig. 2). We identified 1147 differentially expressed genes (DEGs), of which 916 were upregulated and 231 downregulated in resistant possums.

Data from resistant and susceptible possums were clearly distinguished in hierarchical clustering of gene expression, and this also reveals within-group differences in gene expression among upregulated genes. (Supplementary Fig. 5). Examination of the top twenty upregulated and downregulated genes (Supplementary Figs. 6 and 7) illustrates the expression differences between resistant and susceptible possums. It also reveals variation in expression levels among the susceptible possums sampled in New Zealand for some genes such as atp5g3 (ATP Synthase Membrane Subunit C Locus 3), Cox7b (Cytochrome C Oxidase subunit 7B) and CYP8B1 (Cytochrome P450 Family 8 Subfamily B Member 1). These are genes associated with energy metabolism and the metabolism of xenobiotic compounds that might include artificial toxins.

**Fig. 3:**
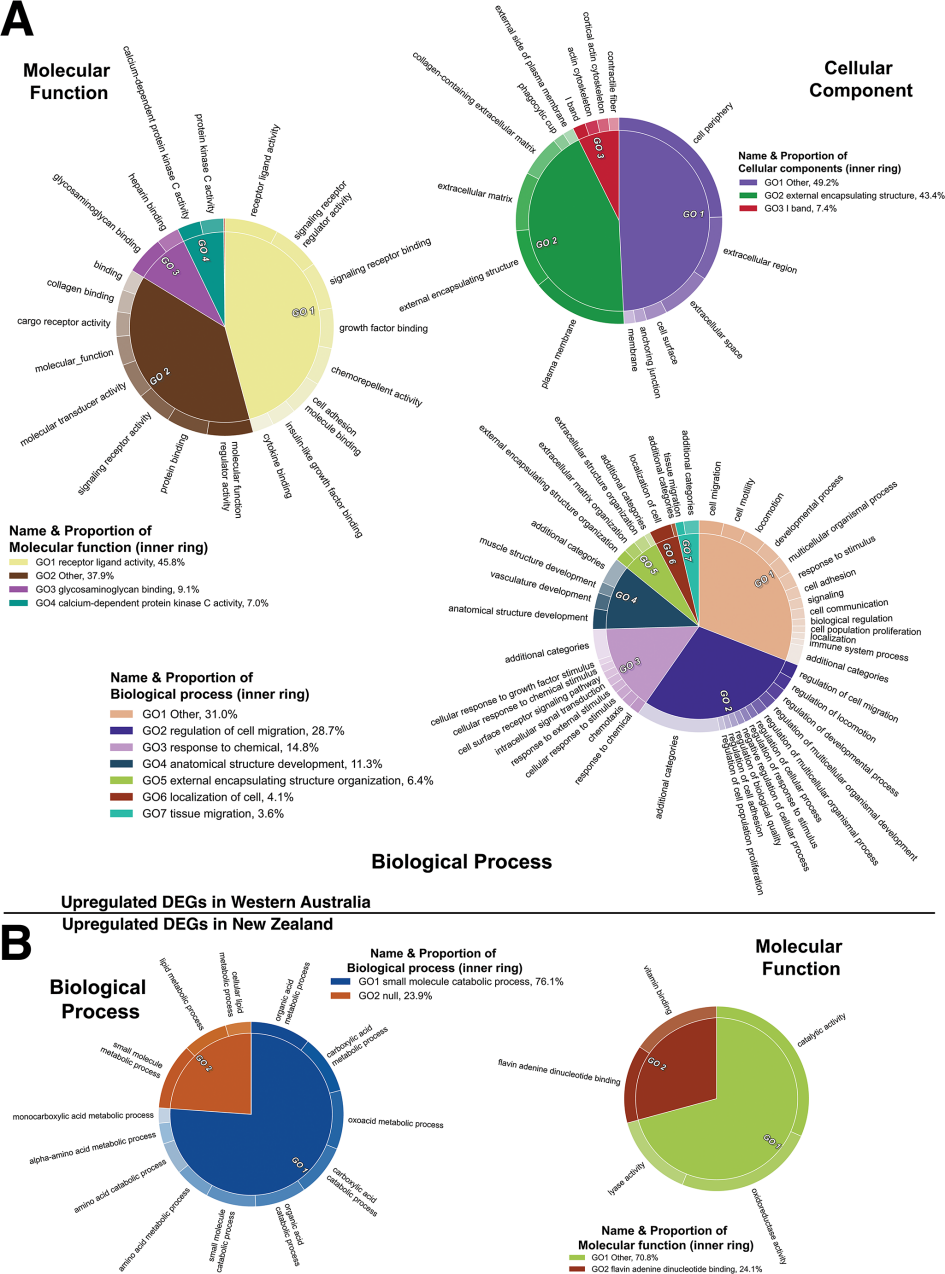
Gene Ontology (GOs) enrichment analysis among genes identified using DESeq2 that were significantly upregulated in (**A**) resistant and (**B**) susceptible brushtail possum (*Trichosurus vulpecula*) liver. Terms are grouped by hierarchical clustering. Parent terms are identified in the legend and their respective proportions, directly proportional to statistical significance. GO terms were first summarized based on a semantic similarity of 0.4 using REVIGO and visualized in CirGO. Circles correspond to one ontology group (BP: Biological process, MF: Molecular function and CC: Cellular component)

Among the identified 1147 differentially expressed genes (DEGs), 133 were genes also found exhibiting significant differential expression associated with development and 55 were rRNA genes (Supplementary Table 1). The first set most likely comes from age variation and might indicate mis-classification of juveniles as adult. The rRNA gene expression variation could be associated with differences in library preparation. These 188 genes were excluded in subsequent analyses, conservatively reducing the DEG set to 959 genes. Among these, gene ontology enrichment analysis revealed 287 enriched terms, 268 among upregulated and 19 among downregulated genes in resistant possums. The Revigo (Supek et al. 2011) grouping of the enriched terms visualized using CirGO (Kuznetsova et al. 2019) showed that a majority of these genes upregulated in resistant possums were associated with the biological processes of *regulation of cell migration* and *anatomical structure development* and the *cellular components of the cytosolic ribosomes* and *external encapsulating structures* (Fig. 3). On the other hand, the downregulated genes in resistant possums are associated with *small molecule catabolic process* and *flavin adenine dinucleotide binding* (Fig. 3).

**Fig. 4:**
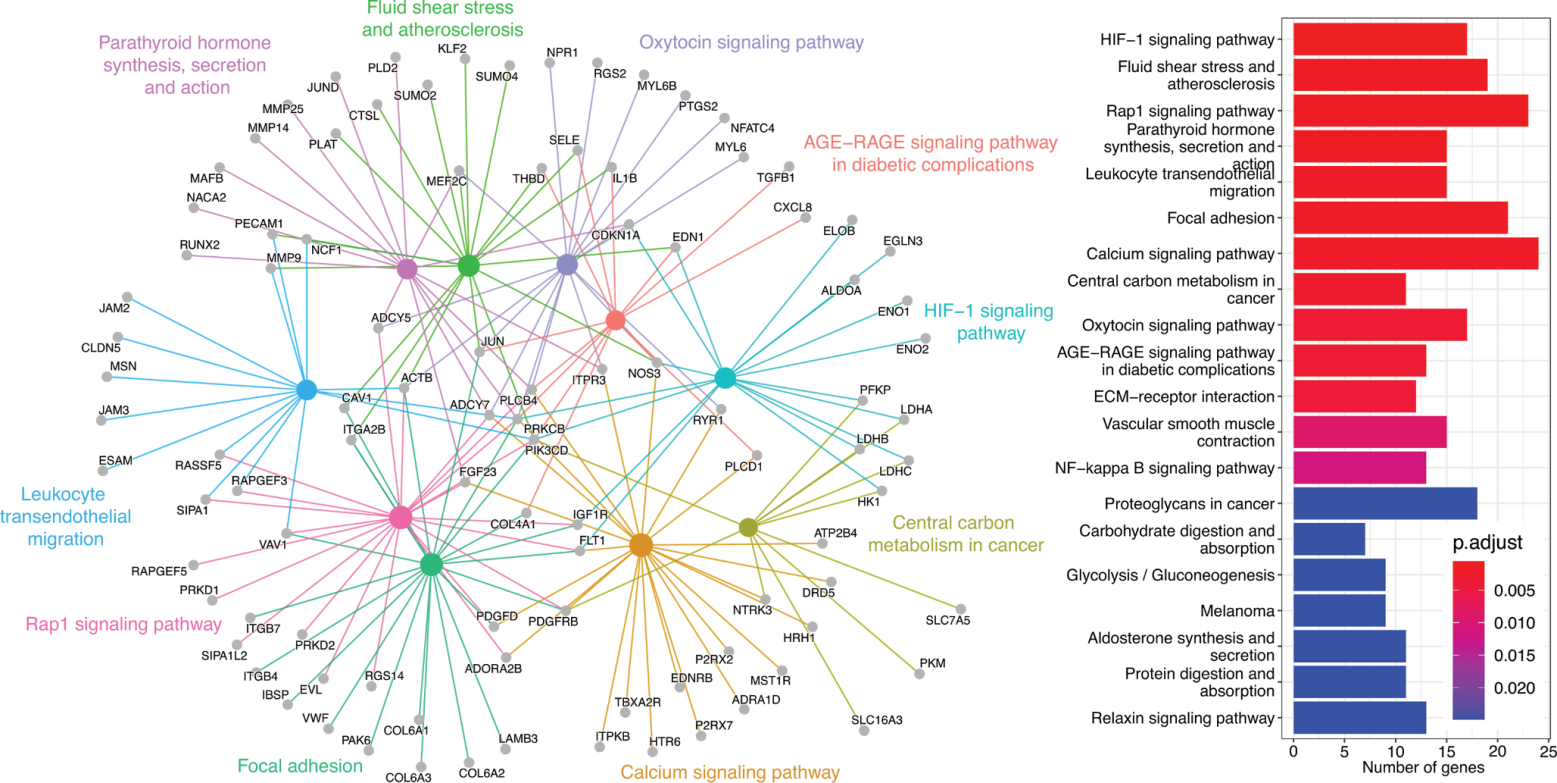
Enrichment by pathway terms visualized using the cnetplot function from the “enrichplot” R package. Significantly enriched KEGG pathways (FDR ≤ 0.05) associated with the significant upregulated DEGs of 1080-resistant brushtail possums (*Trichosurus vulpecula*). Big and coloured nodes represent the pathways and grey nodes are the differentially expressed genes associated with those pathways. The number of genes and FDR-adjusted p-values associated with each significantly enriched pathway are reported on the bar plots

**Fig. 5:**
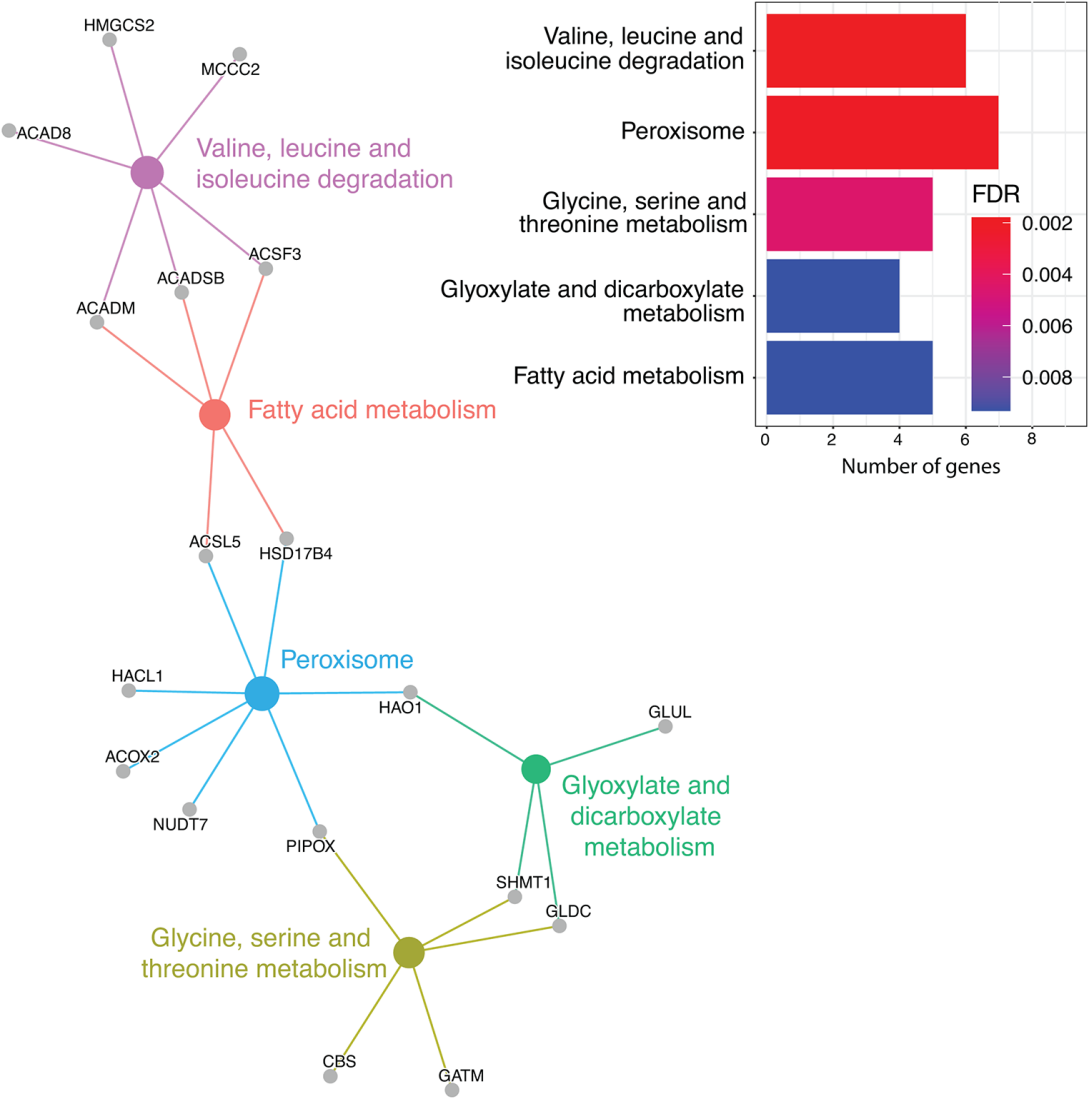
Enrichment by pathway terms visualized using the cnetplot function from the “enrichplot” R package. Significantly enriched KEGG pathways (FDR ≤ 0.05) associated with the significant downregulated DEGs of 1080-resistant brushtail possums (*Trichosurus vulpecula*). Big and coloured nodes represent the pathways and grey nodes are the differentially expressed genes associated with those pathways. The number of genes and FDR-adjusted p-values associated with each significantly enriched pathway are reported on the bar plots

Significantly enriched KEGG pathways associated with the significantly upregulated DEGs in resistant possums are associated with several signalling pathways (*HIF-1*,* Rap1*, *AGE_RAGE*,* Calcium and Oxitocyne*), *focal adhesion* and *carbon metabolism* (Fig. 4). This is consistent with the GO enrichment analysis but adds detail on the possible functions associated with the significant upregulated DEGs in resistant possums. KEGG pathways found among the significantly downregulated DEGs in resistant possums were also consistent with the GOs analysis by displaying *small molecule metabolism pathways*, but also indicated seven genes associated with the *peroxisome* (Fig. 5).

Two other approaches (linear models and correlation networks) were used to compute significantly differentially expressed genes. The Limma package identified 1221 differentially expressed genes (1073 upregulated and 148 downregulated in resistant possums). Those genes allowed us to identify 325 enriched GO terms (Supplementary Fig. 8) that closely match the terms identified from the DEGs identified with DESeq2. The common enriched terms among upregulated DEGs are associated with the *regulation of cell migration*, *chemotaxis* and *external encapsulating structure*. Among downregulated DEGs in resistant possums are *small molecule catabolic processes* (Supplementary Fig. 8). Differential expression analysis using the WGCNA package that considers modules (clusters) of co-expressed genes (Langfelder and Horvath 2008) identified three modules significantly associated with resistant brushtail possums compared to susceptible ones (Supplementary Fig. 9A); two of them positively correlated (Module A and C). Within those modules, the genes having both gene significance and module membership over 0.8 were defined as differentially expressed (Supplementary Fig. 9B). Overall, the WGCNA package identified 1006 differentially expressed genes; two downregulated and 1004 upregulated in resistant possums. This led to finding enriched gene ontology (GO) terms only associated with upregulated genes in resistant possums.

As with results from Limma, many common terms were found in the enriched GOs analysis of the DEGs using the WGNA and the DESeq2 packages, but their ranking of importance differed (Supplementary Fig. 10). Among the additional GO terms discovered among the WGCNA DEGs, many were associated with *G protein-coupled receptor signalling pathway* being the first category of GO term within Biological processes (Supplementary Fig. 10). 64% of differentially expressed genes were discovered by more than one approach in the present analysis (Supplementary Fig. 11), but each package identified a similar proportion of unique differentially expressed genes (DESeq2: 233 unique DEGs, Limma: 173 unique DEGs, WGCNA: 190 unique DEGs). Comparing the respective enriched GO terms, the correlation network approach (package WGCNA) yielded fewer terms than the other two approaches, but each analysis shared more than half of their discovered enriched GO terms with another analysis.

### Carbon metabolism pathways are prominent in the differential expression pattern

**Fig. 6:**
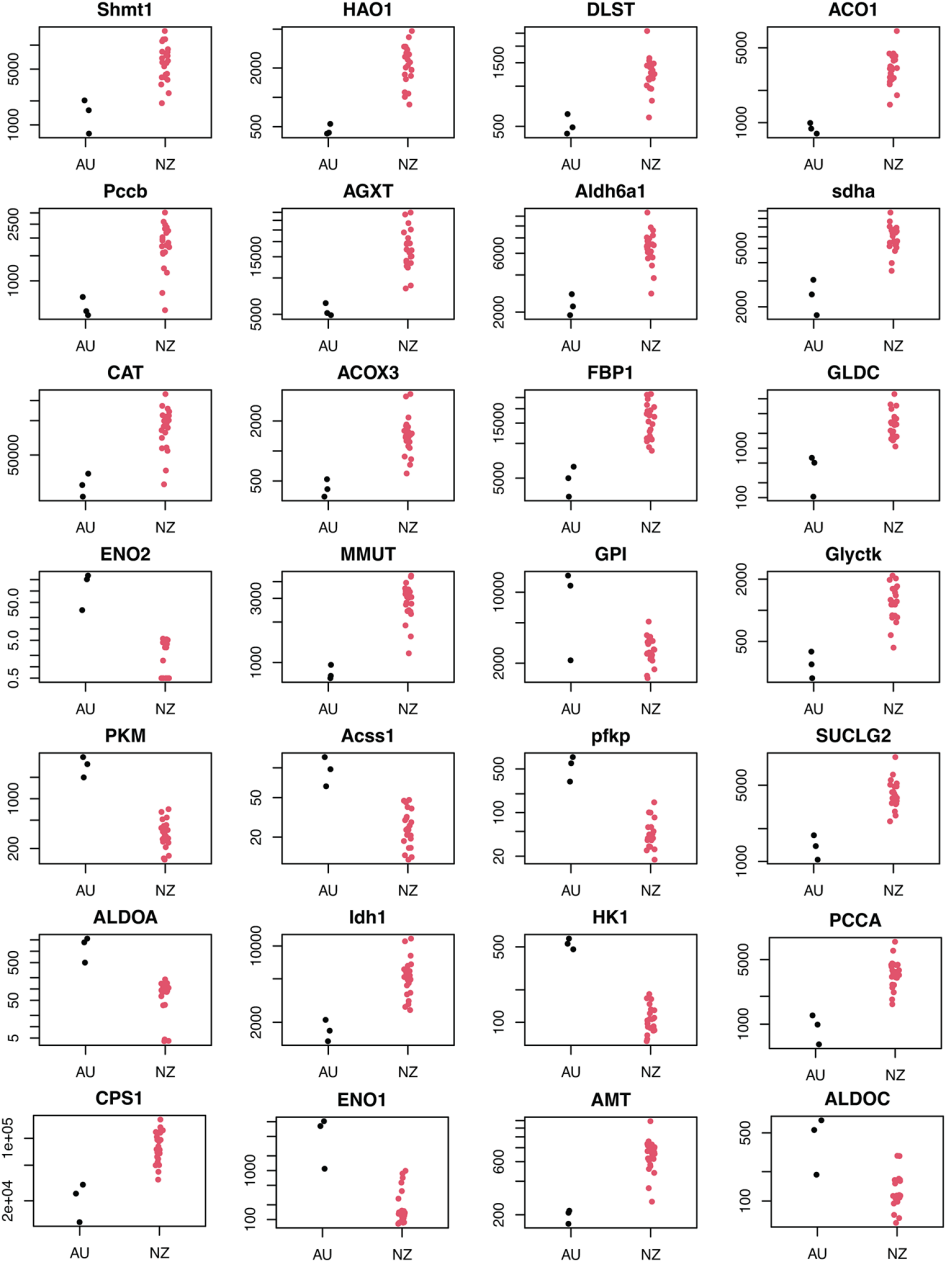
Carbon metabolism” genes differentially expressed in resistant (black) and susceptible brushtail possums (red). Each dot corresponds to the normalized counts of the differentially expressed genes for each liver RNASeq sample (Log-Fold Change <-2 or > 2 & FDR-adjusted p-value < 0.00001)

Enriched KEGG pathways, identified in both upregulated DEGs (Fig. 4) and downregulated DEGs (Fig. 5), consistently include pathways associated with carbon metabolism (central carbon metabolism in cancer, carbohydrate digestion and absorption, glycolysis/gluconeogenesis, glycoxylate and decarboxylate metabolism and fatty acid metabolism). Carbon metabolism is a major component in metabolism of sodium fluoroacetate. The genes associated directly with “carbon metabolism” pathway (KEGG: ko01200) also show obvious differential expression (FDR corrected p-value < 0.00001 & LFC < -2 or > 2), with some upregulated (9) and others downregulated (19) in resistant possums (Fig. 6).

## Discussion

Brushtail possums are arboreal herbivores that, across their geographic range, eat local plants in ecologically distinct regions of Australia (Kerle 1984). The food plants available to them in these environments have different properties, most prominently with high levels of defence chemicals in the flora of Western Australia (Leong et al. 2017). We applied differential gene expression analysis using liver tissue to compare two populations of brushtail possums; one (*T. v. hypoleucus*) that displays elevated resistance to the potent, naturally occurring, mammal toxin sodium fluoroacetate (Twigg et al. 1996; Twigg and King 1991), and one (*T. v. fuliginosis*/*vulpecula*) that is susceptible to this poison. Our core objective being to identify gene expression differences between them that reflect different natural selection via the defence chemicals present in the plants they eat.

### Protected species and power analysis

Given the vulnerable conservation status of brushtail possums in Australia and the need to euthanise for sampling of liver mRNA, the opportunities for sampling this species in their native range were limited. In contrast, there was ample opportunity for sampling this species in New Zealand where it is an invasive pest. Resulting differences in sample size, preparation and sequencing methods that might influence interpretation were addressed using Full-Scale Quantile Normalization, which is designed to correct for batch effects. Additionally, normalization of read number accounts for variation in read depth, avoiding downsampling, which could reduce statistical power without meaningfully addressing platform bias. By applying rigorous thresholds for differential expression (log fold change >|2|; adjusted p-value < 10^-5), and multiple differential expression packages, we maximised confidence in the results. Identifying the minimum number of specimens needed to confidently acquire significant gene expression signal was a crucial step in experimental design. We performed a power analysis using a dense sampling of adults and juveniles from a single population to establish the influence of sample size and p-value threshold on the discovery rate of candidate genes. We found sample size to be important for discovery of a large number of genes that are differentially expressed and to reduce the number of false positives (Conesa et al. 2016; Hart et al. 2013). The GO-term enrichment analysis was quite sensitive to differences in the gene set and especially to potential false positives, however, we found that increasing the number of samples in just one of the two groups being compared was beneficial for the discovery of significant DEGs.

### Resistant and susceptible brushtail possums differ in expression of many genes and pathways

We identified numerous differentially expressed genes of which more than 60% were found with all three of the separate approaches. This level of similarity in the DEGs and enriched GO terms show a consistent biological difference and additional unique genes and pathways identified with each of the three approaches broaden our set of candidate genes. We cannot be certain that all differentially expressed genes (DEGs) identified in this analysis reflect differing degrees of toxin susceptibility, but it is highly likely that a larger proportion are associated with diet. Attributes of food resources are central to growth and survival of animals, and herbivores are entirely dependent on the nutrients sequestered by plants that they eat. As folivore is a major challenge to plant growth and reproductive fitness (Zangerl et al. 2002), herbivores exert strong natural selection on plant metabolite and community diversity (Maron et al. 2019; Speed et al. 2015), and vice versa (Endara et al. 2023). While toxin resistance may not be the only significant factor behind the observed DEG patterns, starkly different toxin resistance has been measured (Twigg et al. 1996; Twigg and King 1991), and by targeting mRNA expression in the liver we have enriched for pathways associated with this organ, which are primarily digestion and detoxification (Grant 1991; McArthur et al. 1991). Furthermore, though sampling from distinct subspecies might imply a broad scale of functional genomic differences, we found most genes (93%) did not differ significantly. From a total of 17,070 discovered genes, the majority (15,923) did not show significant expression difference, yielding a small fraction of candidate genes for further investigation to confirm associations with known physiological differences between the populations.

In considering functional properties of the differentially expressed genes from mRNA in possum liver samples, five major classes were detected in possums:

1. Gene ontology (GO) terms related to *glycosaminoglycan binding*, *ECM-receptor interaction*, and *proteoglycans in cancer* were enriched which suggests divergence related to extracellular matrix interactions.
2. GO terms related to *semaphorin receptor binding* (Alto and Terman 2017), *rap1 signalling pathway*, *leucocyte trans-endothelial migration*, *chemotaxis*, and *regulation of cell migration* were enriched suggesting differences among possum populations related to cell signalling and cell migration.
3. The enrichment of GO terms related to *DNA-binding transcription activator activity* and *NF-kappa B signalling pathway* (Baltimore 2009) is as expected with divergent gene expression and response to environmental stressors.
4. The enrichment of GO terms related to *external encapsulating structure organization*, *anatomical structure development*, and *external encapsulating structure* in the liver suggest physiological/morphological adaptation.
5. Finally, several genes and gene families, commonly associated with drought adaptation in mammals were found differentially regulated in our brushtail possum samples (CYP2E, GPX3, SLC family, KCN family) (Rocha et al. 2021).

### Candidate genes and potential pathways for resistance to plant toxins

**Fig. 7:**
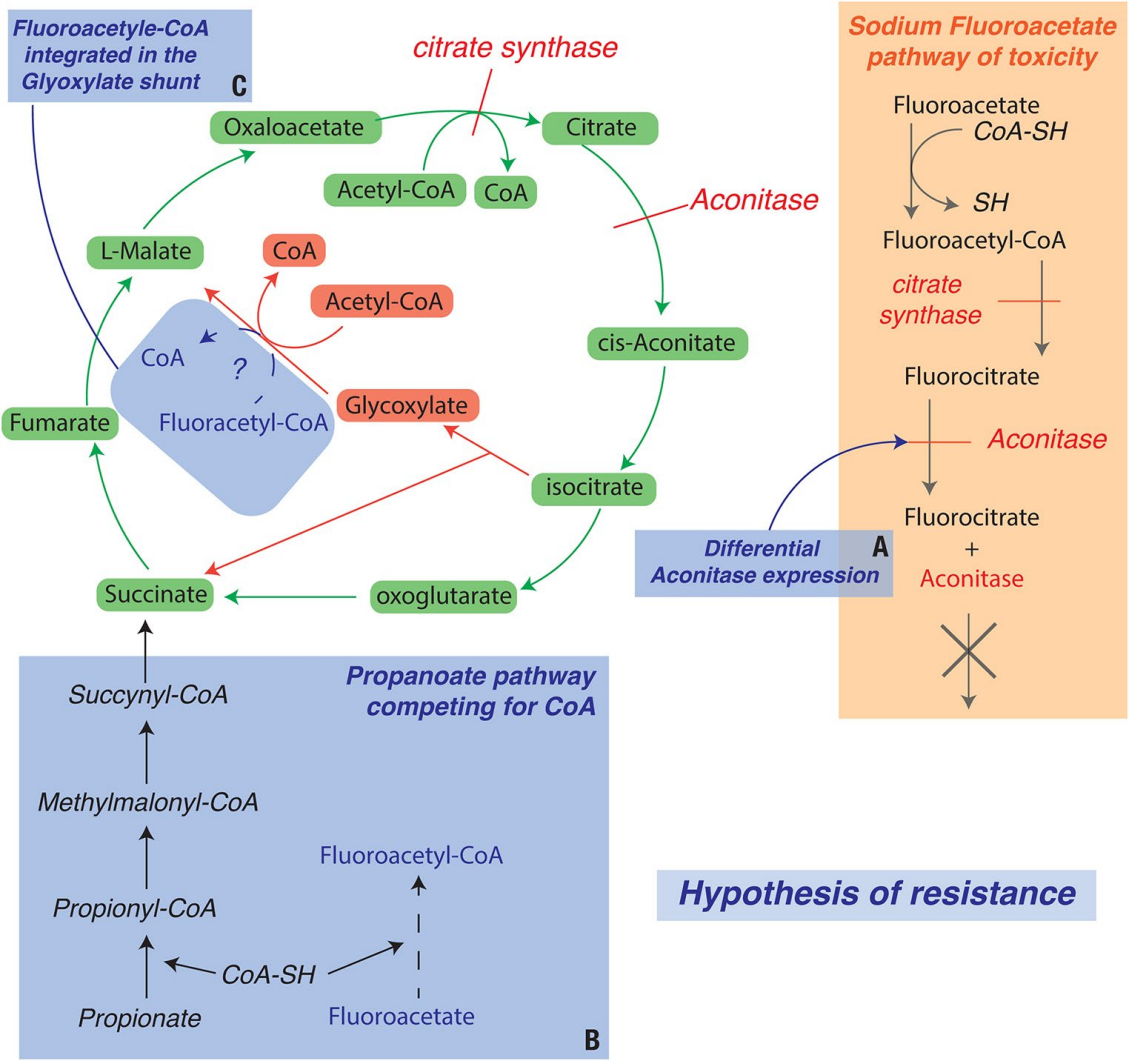
Molecular pathway associated with sodium fluoroacetate (1080) poisoning and hypothesis for resistance associated with the differential expression results from RNA-Seq data from brushtail possum (*Trichosurus vulpecula hypoleucus*) liver. The three main hypotheses of resistance are **A**: an underexpression of the aconitase limiting the pathway of toxicity of sodium fluoroacetate, **B**: the overexpression of the propanoate pathway competing for association with Coenzyme A first necessary step for fluoroacetate toxicity and **C**: the overexpression of glycoxylate shunt pathway used to metabolise fluoroacetyl-CoA avoiding the toxic association with acotinase

It has previously been proposed that toxin resistance in Australian mammals implicates adaptation of the aconitase gene ACO2, but testing this hypothesis using tammar wallaby (*Notamacropus eugenii*) failed to find DNA sequence differences (Deakin et al. 2013). The gene ACO1 is present in our data and displays some signal for overexpression in liver from susceptible possums (Fig. 6, Supplementary Fig. 12, Fig. 7A).

Cellular and metabolic functions of differentially expressed genes form pathways that have been mapped to produce networks of molecular interactions, and these are linked to gene functions and products within the KEGG database. KEGG pathway enrichment provided an opportunity to identify candidate genes for resistance to plant toxins. The toxin sodium fluoroacetate (1080) interferes with the tricarboxylic acid (TCA) cycle by inhibiting the enzyme aconitase. This inhibition leads to a build-up of fluorocitrate that cannot be converted to oxaloacetate by the aconitase, depleting cellular energy stores and ultimately causing death (Goncharov et al. 2006). Examination of the KEGG pathway enrichment analysis on all genes with adjusted p-value under 0.00001 shows a strong association with carbon metabolism with DEGs within pathways such as *metabolism of Glyoxylate and dicarboxylate*, *Carbon*, and *Propanoate*. The differential expression of these pathways suggests candidates for involvement in resistance to sodium fluoroacetate, and closer examination provides some support for this hypothesis.

- *Propanoate metabolism*: One potential treatment of sodium fluoroacetate poisoning is using competing substances to bind with coenzyme A (CoA) such as propanoate (Goncharov et al. 2006). The propanoate metabolism pathway comprises all the genes associated with incorporating propanoate into the TCA and the first step consists of propanoate to propionyl-CoA binding the molecule to CoA (Fig. 7B), In our analyses several genes associated with this pathway showed significant differential expression (LDHA, LDHB, LDHC, PCCA, PCCB, ALDH6A1, ACSS1…).
- *Glyoxylate and dicarboxylate metabolism*: The glyoxylate cycle bypasses the CO_2_-generating steps of the TCA cycle and allows synthesis of malate from glyoxylate using malate synthase (Fig. 7C). It has been suggested that this pathway could be upregulated in organisms that are resistant to sodium fluoroacetate, allowing them to use fluoroacetyl-CoA instead of acetyl-CoA to synthesise malate (fluoromalate) (Marletta et al. 1981; Powell and Beevers 1968).
- The genes ENO1, ENO2, PKM, PFKP, ALDOA, and HK1 are all strongly upregulated in resistant samples and encode for enzymes involved in the glycolytic pathway. Enolase (ENO) catalyses the conversion of 2-phosphoglycerate to phosphoenolpyruvate, and pyruvate kinase (PKM) catalyses the conversion of phosphoenolpyruvate to pyruvate, generating ATP in the process. Phosphofructokinase (PFKP), aldolase A (ALDOA) and hexokinase 1 (HK1) are also involved in the glycolysis. The upregulation of these genes in resistant individuals could be associated with a shift in energy production towards glycolysis, bypassing the TCA cycle and its inhibition by the sodium fluoroacetate.
- ACSS1 gene is upregulated in the liver of resistant possums. ACSS1 codes for acetyl-CoA synthetase, an enzyme that is involved in the production of acetyl-CoA and is also involved in the synthesis of fluoroacetyl-CoA the first step of sodium fluoroacetate intoxication. This upregulation could mean that either fluoroacetyl-CoA is dealt with as we hypothesize through the glyoxylate shunt or that this version of the gene is different in the resistant possums and could synthesise acetyl-CoA faster than fluoroacetyl-CoA avoiding the inhibition of aconitase and the accumulation of fluorocitrate.

Overall, the number and combination of genes differentially regulated and associated with the TCA cycle leads us to suggest that regulation of. this pathway is a strong candidate for direct involvement with sodium fluoroacetate resistance observed in brushtail possums from Western Australia (Fig. 7). It is unlikely that resistance in possums is controlled by a single gene but, like many physiological adaptations (Reid et al. 2023; Thornton 2019), is the result of the combined effects of multiple genes as regulation of the TCA cycle is a highly polygenic trait (Barghi et al. 2020; Margres et al. 2017).

In order to limit potential bias associated with different developmental stage in our samples we decided to identify and remove differentially expressed genes between adult and juvenile possums. However, the cytochrome P450 (CYP) family, known for its role in metabolizing toxic compounds, differentially expressed between adult and juvenile possums warrants specific mention due to its relevance to the possums’ diet and toxin resistance. Cytochrome P450s genes code for membrane-bound proteins that catalyse the metabolism of a diverse array of xenobiotic compounds and endogenous substrates, they are critically important in detoxifying and eliminating drugs, chemicals and environmental pollutants. In mammals, various CYPs also participate in the biosynthesis and metabolism of steroids (El-Merhibi et al. 2011; Seliskar and Rozman 2007). Increased expression in adult possums compared to juveniles likely reflects the change of diet during weaning from maternal milk to plant leaves (Cowan 1989; How and Hillcox 2000; Tyndale-Biscoe 2005). A similar increase of CYPs (P450s) expression during development of mice has been detected in a similar way (Peng et al. 2012). These genes deserve further study that focuses on toxin resistance displayed by Western Australian possum subspecies (Twigg et al. 2003) and knowing that their expression varies during development adds extra evidence of their possible association with the diet of the possums (Bond et al., 2023). Careful experiment design will be needed for the study of adaptive shifts in cytochrome P450s as expression levels could be confounded with different maturity levels of the possum samples rather than adaptations to local diet.

## Conclusion

These data contain the basis for inferring a potential biochemical pathway to increased sodium fluoroacetate (1080) resistance that specifically informs understanding of brushtail possum biology and furnishes the basis for targeted analyses to test the role of several candidate genes. One approach to investigate this novel hypothesis will be the use of qRT-PCR tools (Bustin et al. 2005) to measure in individuals of known exposure the relative expression among candidate and housekeeping genes. Understanding this adaptation will have applied benefits for mammal conservation and predator control in Australia, where 1080 is widely applied against introduced species (Eason et al. 2011), and in New Zealand where the same toxin is used against brushtail possums (Alterio 2000; Innes and Barker 1999; Ross 1999). As a system for exploration of mammal-plant coevolution (Freeland 1991; Tucker et al. 2010) *Trichosurus vulpecula* is exceptional as it simultaneously displays natural environmental response gradients in Australia whilst being subject to large scale and repeated exposure to a synthetic analogue of a key plant toxin in what amounts to a nationwide selection experiment in New Zealand.

## Electronic supplementary material

Below is the link to the electronic supplementary material.

Supplementary Material 1

Supplementary Material 2

## Data Availability

Tables of read counts and gene-associated p-values for DESeq2, limma and WGCNA analysis, the list of significant differential expressed genes, the tables of significant GO terms and the associated R script can be found on the data repository during review: https://figshare.com/s/476983506de4ee6c33c6. Associated DOI will then be generated prior to publication. Sequences can be found on the NCBI SRA database associated with the Bioprojects PRJNA1083711 (data produced by the authors) and PRJNA323970 (data produced by Tim Hore Lab: (Bond et al., 2023).
