# Supplementary material for: Metabolic differentiation of brushtail possum populations resistant and susceptible to plant toxins revealed via differential gene expression
Source: J Comp Physiol B. 2024 Nov 4;195(1):103–21. doi: 10.1007/s00360-024-01591-z (PMC11839783; doi:10.1007/s00360-024-01591-z)
Supplement: Supplementary file 1 — Supplementary Material 1 [file 360_2024_1591_MOESM1_ESM.pdf]

How much does sample size limit DEGs discovery and confidence?

To investigate the influence of sample size on the number of genes identified as differentially expressed, the differential expression and the gene ontology enrichment analyses were run again using different subsets of samples and different threshold p-values. A flowchart representing three different algorithms is available in Figure 1. These analyses were done using the same tools and data as from the main article (R Development Core Team, 2021).

1) The first approach used between two and eight randomly selected samples in each group (juveniles and adults). For example, three randomly selected juveniles and three randomly selected adults. The selected subset of samples was then analysed in a differential expression and gene ontology enrichment analysis as described above (with similar p-value and fold change thresholds). The significant DEGs and significantly enriched GO terms were then compared to the one issued from the analysis of the whole dataset. The proportion of common significant DEGs and significantly enriched GO terms are then retained. For each sample subset size, the algorithm was run twenty times (total number of runs = 140).
2) A modification of this analysis used a sample subset size that could take any of all possible values (juveniles 2 to 9, adults 2 to 14), the significantly enriched GO terms are not recorded and the number of iterations for each combination is reduced to ten (total number of runs = 1040).
3) Finally, the influence of the p-value threshold was studied using the same model with subset sizes from two to nine random samples and the threshold of significance for the DEGs varying between four different p-values (p-value < 0.01, 0.001, 0.0001 and 0.00001). Ten iterations per combination were used (total number of runs = 320).

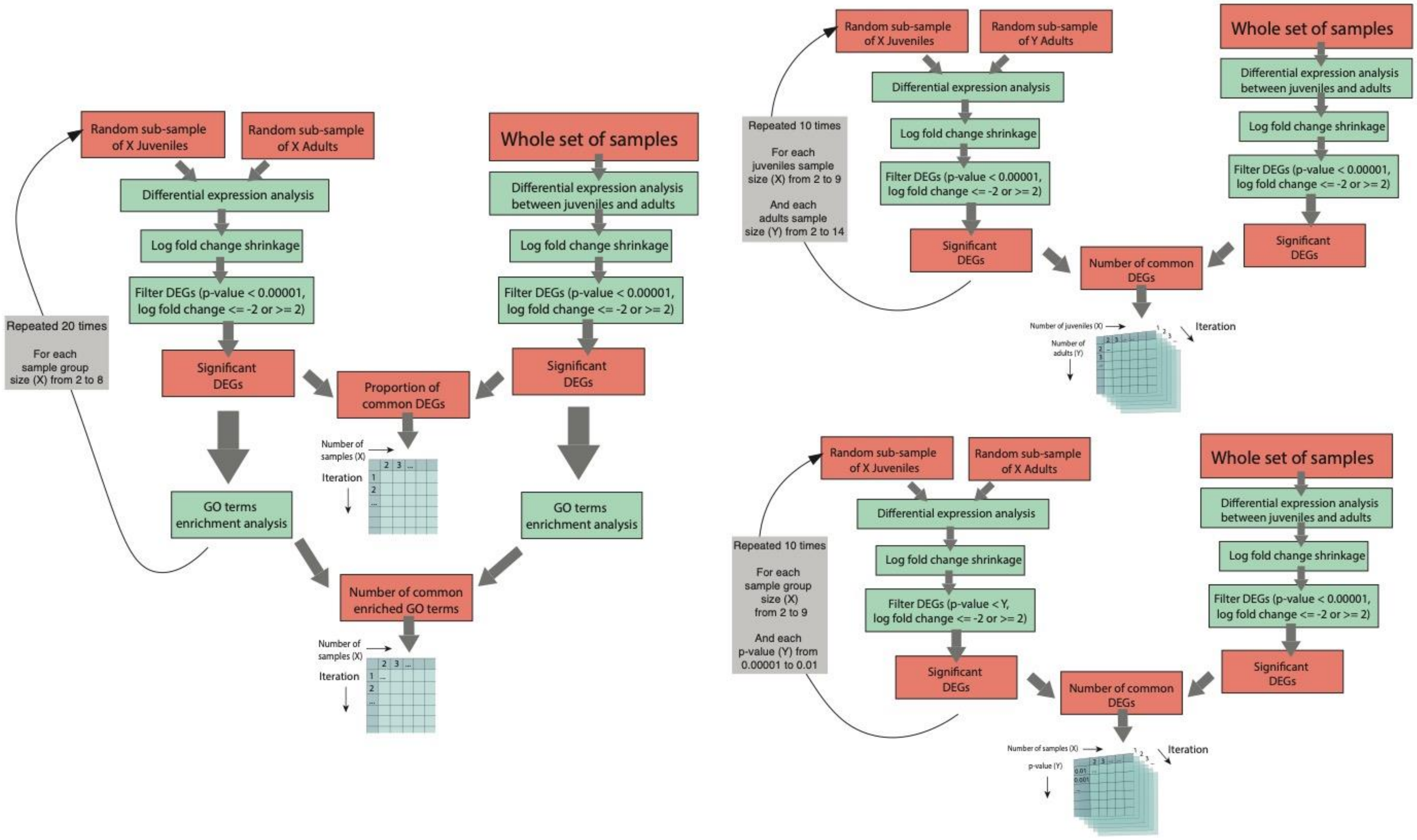

Figure 1: Flowchart of the three different power algorithms.

The first results show a significant influence of sample size on the proportion of common discovered genes with a median starting at less than 20% for 2 samples in each group and reaching around 80% for groups of 8 samples (Figure 2, A). The increasing number of common discovered genes seems to come with reduced variance and be consistent with the increase in sample size, the biggest gap seems to correspond with the group size from 2 to 3 samples (Figure 2, A). The reduced variance corresponds to the subset of the sample being more similar to each other with the increased sample size.

Another result to mention is the similar evolution of common discovered enriched GOs (Figure 15, B) but the variance is very high for group sizes of 3, 4 and 5 with the number of common GOs varying from 0% to 85%. Similarly, to the common DEGs, the variance is reducing with the number of samples per group and as the number of common GOs terms is increasing (Figure 24, B).

The threshold p-value seems also to be crucial when recovering DEGs, there is a clear increase of common DEGs with an increased maximum p-value. For 2 samples per group increasing the p-value allows the proportion of common DEGs to reach almost 50%. This increase in common DEGs is less marked with a higher number of samples, from more than 25% to around 10% (Figure 2, C). A side effect of the increase of the maximum p-value for DEGs significance is an increase in the false discovery rate, this rate represents the proportion of significant DEGs that are not considered significant when looking at the full samples set and with a p-value of 0.00001. This false discovery rate also reduces with the number of samples but never reaches more than 26% of the number of discovered DEGs.

It is very interesting to notice how many common DEGs are recovered with increasing the number of samples in only one group (Figure 2, D), for example with 4 juveniles and 2 adults the mean of ten runs reaches 26% of common significant DEGs when 4 juveniles and 14 adults the value is slightly less than 63%.

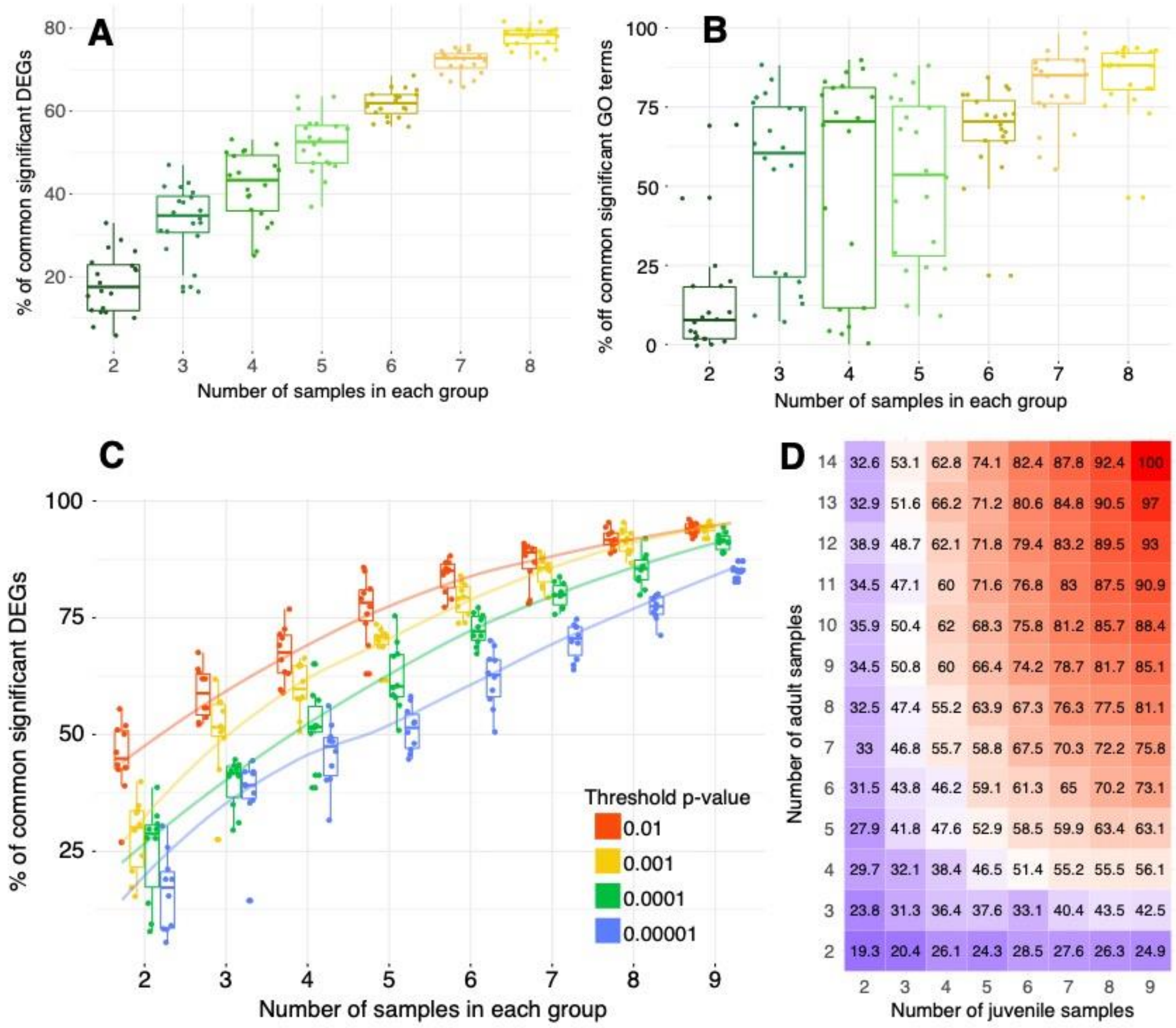

Figure 24: A) Proportion of common significant DEGs (differentially expressed genes) between the results from differential expression analysis on the whole set of samples (9 and 14 adults juveniles brushtail possums livers samples) and a subset of samples with a set number of samples. **B)** Proportion of common significant GO terms (Gene ontology) between the results of GO enrichment analysis from differential expression analysis and on the whole set of samples (9 juveniles brushtail possums and 14 adults) and a subset of samples with a set number of samples. **C)** Proportion of common significant DEGs (differentially expressed genes) between the results from differential expression analysis on the whole set of samples (9 juveniles brushtail possums and 14 adults) and a subset of samples with a set number of samples and a different set p-value. **D)** Mean proportion in % (from 10 runs) of common significant DEGs between the results from differential expression analysis on the whole set of samples (9 juveniles brushtail possums and 14 adults) and a subset of samples (randomly chosen).
