# Supplementary material for: Metabolic differentiation of brushtail possum populations resistant and susceptible to plant toxins revealed via differential gene expression
Source: J Comp Physiol B. 2024 Nov 4;195(1):103–21. doi: 10.1007/s00360-024-01591-z (PMC11839783; doi:10.1007/s00360-024-01591-z)
Supplement: Supplementary file 2 — Supplementary Material 2 [file 360_2024_1591_MOESM2_ESM.docx]

Supplementary Material: Metabolic differentiation of brushtail possum populations resistant and susceptible to plant toxins revealed via differential gene expression.

David Carmelet-Rescan*, Mary Morgan-Richards, Steven A. Trewick

Wildlife and Ecology, School of Natural Sciences, Massey University, Private Bag 11-222, Palmerston North, New Zealand.

*Corresponding author: David Carmelet-Rescan. email: [dcarmelet@gmail.com](mailto:dcarmelet@gmail.com)

**ORCID ID:**

David Carmelet-Rescan: 0000-0002-9524-7588

Mary Morgan-Richards: 0000-0002-3913-9814

Steven A. Trewick: 0000-0002-4680-8457

**Supplementary Fig. 1:** Flowchart of the complete method of the study of gene expression in liver of brushtail possums.

** Supplementary Fig. 2**: Number and assignment of reads from RNASeq for each adult possum liver sample in the differential expression analysis. “Assigned” corresponds to read that mapped once to an annotated genome region of the reference. “Multi-mapping” corresponds to reads that map to several places in the reference genome. “No features” are reads that map to the genome in regions without gene annotation, and “Unmapped” are reads that did not map to the reference brushtail possum genome.

**
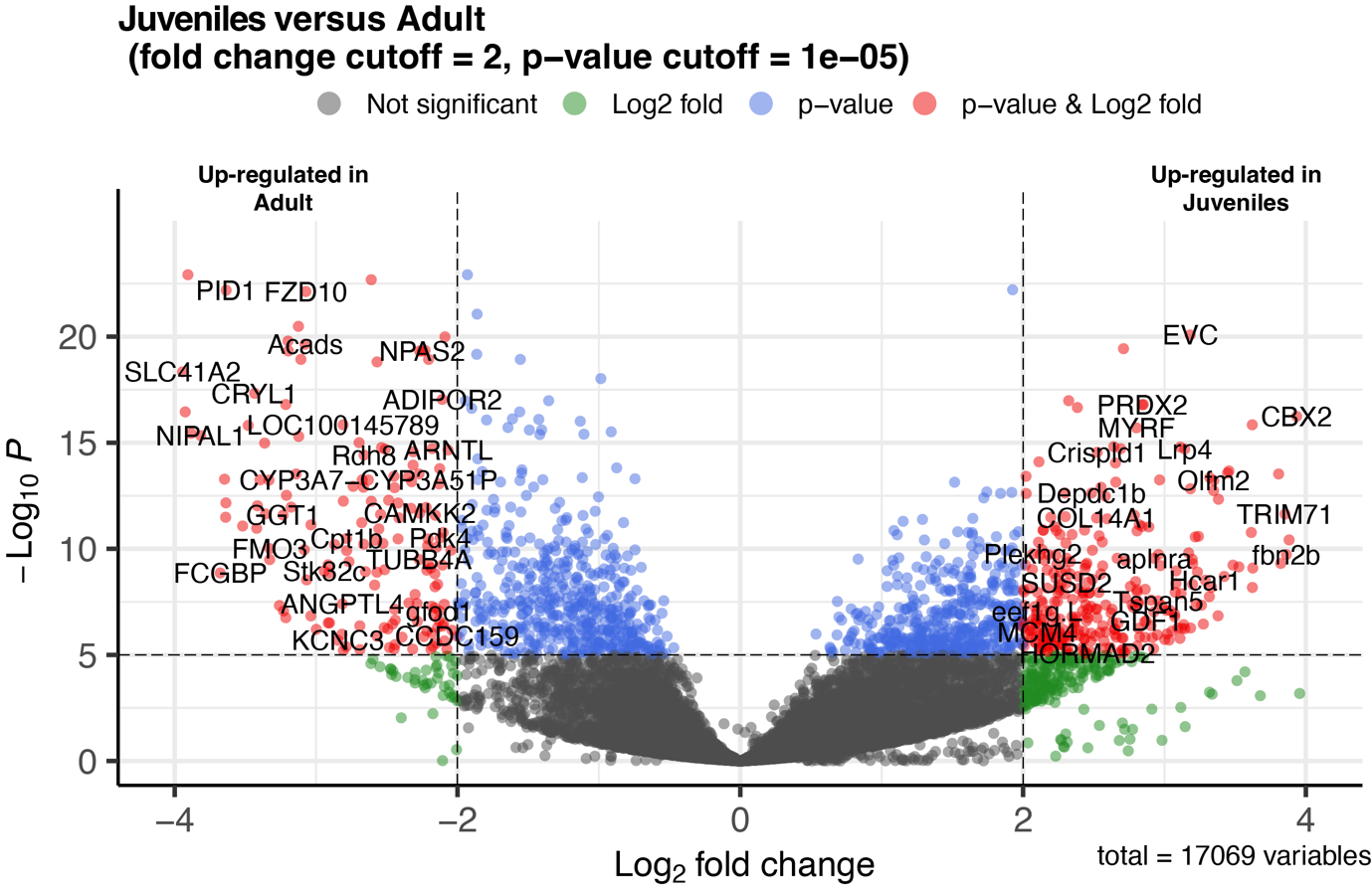
**

**Supplementary Fig. 3:** Visualization of differentially expressed genes (DEGs) from brushtail possums (*Trichosurus vulpecula)* liver samples using volcano plots in R package “DESeq2”. The plot compared the DEGs between adults and juveniles. The representations are as follows: x-axis, log2 fold change; y-axis, -log10 of a p-value. The p-values < 0.00001 are in blue dots, and logFC ≥ 2 and logFC ≤ –2 are green dots; the significant DEGs that satisfy both values are red dots and indicated with gene names. Black dots indicate the majority of genes present in the array that did not differ significantly in expression levels between adults and juveniles. The genes that are upregulated in the liver of juveniles compared to adults are on the right panel, and downregulated ones are on the left panel of the plot.


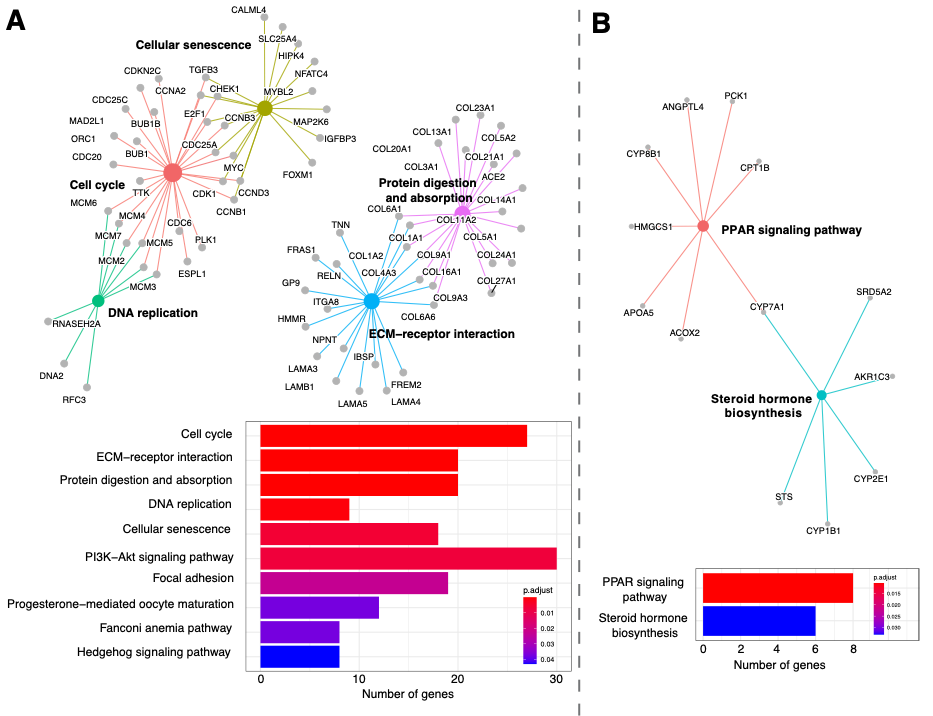


**Supplementary Fig. 4:** Enrichment by pathway terms is visualized using the cnetplot function from the “enrichplot” R package. Significantly enriched Kegg pathways (p-value ≤ 0.05) associated with the significant DEGs between juveniles and adults brushtail possums (*Trichosurus vulpecula*) RNASeq liver samples, (A: upregulated in juveniles, B: downregulated in juveniles). Big and coloured nodes represent the pathways and grey nodes are the differentially expressed genes associated with those pathways. Nodes are sized using enrichment analysis p-values. The number of genes and FDR-adjusted p-values associated with each significantly enriched pathway are reported on the bar plot.

**Supplementary Fig. 5:** Heatmap representation of 1147 differentially expressed genes (DEGs) between samples from toxin-resistant (Western Australia) and toxin-sensitive (New Zealand) brushtail possum (*Trichosurus vulpecula*) liver samples using R packages “DESeq2”. Rows represent individual genes, columns represent samples, and the colour intensity represents the expression levels of each gene in each sample. Sample origin is indicated with the colour band (Green: Western Australian, Orange: New Zealand). The heatmap clusters groups together based on similar expression patterns and highlights any differences between samples based on gene expression

**Supplementary Fig. 6:** Normalized counts of the 20 most significantly downregulated genes in liver samples from toxin-resistant (western Australian) compared to toxin-susceptible (New Zealand) brushtail possums (*Trichosurus vulpecula*) RNASeq data. 1080-toxin-resistant black; 1080-toxin-susceptible red.

**Supplementary Fig. 7:** Normalized counts of the 20 most significantly upregulated genes in the liver of toxin-resistant (western Australian) compared to toxin-susceptible (New Zealand) brushtail possums (*Trichosurus vulpecula*) RNASeq samples. Toxin-resistant are coloured black and toxin-susceptible possums are red.

**Supplementary Table 1**: List of genes removed prior to Genes Ontology and KeGG analysis of differential gene expression (rRNA and genes common to development differential expression analysis)

**Gene Full name**

CYFIP2 cytoplasmic FMR1 interacting protein 2(CYFIP2)

RPL30 ribosomal protein L30(RPL30)

SPARC secreted protein acidic and cysteine rich(SPARC)

RPL32 ribosomal protein L32(RPL32)

RPL31 ribosomal protein L31(RPL31)

RASL11A RAS like family 11 member A(RASL11A)

MSMO1 methylsterol monooxygenase 1(MSMO1)

KLHDC7A kelch domain containing 7A(KLHDC7A)

SLC4A1 solute carrier family 4 member 1(SLC4A1)

RPL6 ribosomal protein L6(RPL6)

AMOT angiomotin(AMOT)

RPS15 ribosomal protein S15(RPS15)

RPS14 ribosomal protein S14(RPS14)

EFEMP2 EGF containing fibulin extracellular matrix protein 2(EFEMP2)

STS steroid sulfatase(STS)

RPS16 ribosomal protein S16(RPS16)

RPL18A ribosomal protein L18a(RPL18A)

RPL36AL ribosomal protein L36a like(RPL36AL)

RGS1 regulator of G protein signaling 1(RGS1)

RPS18 ribosomal protein S18(RPS18)

CREB3L1 cAMP responsive element binding protein 3 like 1(CREB3L1)

MYC MYC proto-oncogene, bHLH transcription factor(MYC)

STMN1 stathmin 1(STMN1)

RPS6KA1 ribosomal protein S6 kinase A1(RPS6KA1)

TUBB1 tubulin beta 1 class VI(TUBB1)

RPL36 ribosomal protein L36(RPL36)

SLC16A6 solute carrier family 16 member 6(SLC16A6)

RPL35 ribosomal protein L35(RPL35)

RPL38 ribosomal protein L38(RPL38)

SLC26A10 solute carrier family 26 member 10(SLC26A10)

PHLDA2 pleckstrin homology like domain family A member 2(PHLDA2)

RPL39 ribosomal protein L39(RPL39)

HMGN3 high mobility group nucleosomal binding domain 3(HMGN3)

RPS10 ribosomal protein S10(RPS10)

FADS1 fatty acid desaturase 1(FADS1)

SLC16A5 solute carrier family 16 member 5(SLC16A5)

RPS12 ribosomal protein S12(RPS12)

RPS7 ribosomal protein S7(RPS7)

RPL21 ribosomal protein L21(RPL21)

UNC5A unc-5 netrin receptor A(UNC5A)

RPS5 ribosomal protein S5(RPS5)

IGFBP3 insulin like growth factor binding protein 3(IGFBP3)

CACNA2D1 calcium voltage-gated channel auxiliary subunit alpha2delta 1(CACNA2D1)

FIBCD1 fibrinogen C domain containing 1(FIBCD1)

RPS6 ribosomal protein S6(RPS6)

RPL22 ribosomal protein L22(RPL22)

PASK PAS domain containing serine/threonine kinase(PASK)

NPNT nephronectin(NPNT)

RUNDC3A RUN domain containing 3A(RUNDC3A)

CPT1B carnitine palmitoyltransferase 1B(CPT1B)

PDGFRL platelet derived growth factor receptor like(PDGFRL)

SIGIRR single Ig and TIR domain containing(SIGIRR)

RBP7 retinol binding protein 7(RBP7)

OLFM2 olfactomedin 2(OLFM2)

ADGRB2 adhesion G protein-coupled receptor B2(ADGRB2)

LACTB2 lactamase beta 2(LACTB2)

RPL24 ribosomal protein L24(RPL24)

RPL27 ribosomal protein L27(RPL27)

RPL29 ribosomal protein L29(RPL29)

TRIM10 tripartite motif containing 10(TRIM10)

RPL28 ribosomal protein L28(RPL28)

PDE9A phosphodiesterase 9A(PDE9A)

PLEKHD1 pleckstrin homology and coiled-coil domain containing D1(PLEKHD1)

IGSF3 immunoglobulin superfamily member 3(IGSF3)

SLC1A1 solute carrier family 1 member 1(SLC1A1)

WNK4 WNK lysine deficient protein kinase 4(WNK4)

C2CD4B C2 calcium dependent domain containing 4B(C2CD4B)

ADRB2 adrenoceptor beta 2(ADRB2)

RPS4Y1 ribosomal protein S4 Y-linked 1(RPS4Y1)

MRPL13 mitochondrial ribosomal protein L13(MRPL13)

NPAS2 neuronal PAS domain protein 2(NPAS2)

MRPL20 mitochondrial ribosomal protein L20(MRPL20)

PRDX2 peroxiredoxin 2(PRDX2)

NPC1L1 NPC1 like intracellular cholesterol transporter 1(NPC1L1)

VILL villin like(VILL)

RIMKLB ribosomal modification protein rimK like family member B(RIMKLB)

S100A10 S100 calcium binding protein A10(S100A10)

RPL41 ribosomal protein L41(RPL41)

SRD5A2 steroid 5 alpha-reductase 2(SRD5A2)

HOMER2 homer scaffold protein 2(HOMER2)

BCL11A BCL11 transcription factor A(BCL11A)

MCAM melanoma cell adhesion molecule(MCAM)

GFPT2 glutamine-fructose-6-phosphate transaminase 2(GFPT2)

CBX2 chromobox 2(CBX2)

SLC16A11 solute carrier family 16 member 11(SLC16A11)

COL1A1 collagen type I alpha 1 chain(COL1A1)

RPS25 ribosomal protein S25(RPS25)

NR4A1 nuclear receptor subfamily 4 group A member 1(NR4A1)

MEX3B mex-3 RNA binding family member B(MEX3B)

COL1A2 collagen type I alpha 2 chain(COL1A2)

DAO D-amino acid oxidase(DAO)

RPS29 ribosomal protein S29(RPS29)

RPL27A ribosomal protein L27a(RPL27A)

COL5A2 collagen type V alpha 2 chain(COL5A2)

CRYL1 crystallin lambda 1(CRYL1)

RPS21 ribosomal protein S21(RPS21)

TUBA8 tubulin alpha 8(TUBA8)

RPS23 ribosomal protein S23(RPS23)

FKBP10 FKBP prolyl isomerase 10(FKBP10)

ACY3 aminoacylase 3(ACY3)

RPLP0 ribosomal protein lateral stalk subunit P0(RPLP0)

GPT glutamic--pyruvic transaminase(GPT)

MRPL34 mitochondrial ribosomal protein L34(MRPL34)

MLN motilin(MLN)

ROBO1 roundabout guidance receptor 1(ROBO1)

RPL7A ribosomal protein L7a(RPL7A)

AOX1 aldehyde oxidase 1(AOX1)

CA9 carbonic anhydrase 9(CA9)

ACADS acyl-CoA dehydrogenase short chain(ACADS)

TINAG tubulointerstitial nephritis antigen(TINAG)

PLEKHG2 pleckstrin homology and RhoGEF domain containing G2(PLEKHG2)

MMP2 matrix metallopeptidase 2(MMP2)

AVPR2 arginine vasopressin receptor 2(AVPR2)

EMP1 epithelial membrane protein 1(EMP1)

RPL13A ribosomal protein L13a(RPL13A)

RPS3A ribosomal protein S3A(RPS3A)

TMC6 transmembrane channel like 6(TMC6)

MRPS6 mitochondrial ribosomal protein S6(MRPS6)

PITPNM3 PITPNM family member 3(PITPNM3)

ANGPTL4 angiopoietin like 4(ANGPTL4)

AHDC1 AT-hook DNA binding motif containing 1(AHDC1)

MEGF6 multiple EGF like domains 6(MEGF6)

DDC dopa decarboxylase(DDC)

MYRF myelin regulatory factor(MYRF)

RPL11 ribosomal protein L11(RPL11)

LRRC17 leucine rich repeat containing 17(LRRC17)

RPL36A ribosomal protein L36a(RPL36A)

ADCY4 adenylate cyclase 4(ADCY4)

LRP4 LDL receptor related protein 4(LRP4)

ABCB11 ATP binding cassette subfamily B member 11(ABCB11)

CYGB cytoglobin(CYGB)

PTHLH parathyroid hormone like hormone(PTHLH)

CIART circadian associated repressor of transcription(CIART)

CAND2 cullin associated and neddylation dissociated 2 (putative)(CAND2)

RPS15A ribosomal protein S15a(RPS15A)

RPL14 ribosomal protein L14(RPL14)

TRIM47 tripartite motif containing 47(TRIM47)

AHSP alpha hemoglobin stabilizing protein(AHSP)

RPS2 ribosomal protein S2(RPS2)

RPL18 ribosomal protein L18(RPL18)

RPL17 ribosomal protein L17(RPL17)

RPL19 ribosomal protein L19(RPL19)

NQO1 NAD(P)H quinone dehydrogenase 1(NQO1)

CDKN2C cyclin dependent kinase inhibitor 2C(CDKN2C)

GDF15 growth differentiation factor 15(GDF15)

RPL35A ribosomal protein L35a(RPL35A)

CYP8B1 cytochrome P450 family 8 subfamily B member 1(CYP8B1)

COL3A1 collagen type III alpha 1 chain(COL3A1)

SPRY2 sprouty RTK signaling antagonist 2(SPRY2)

RPL22L1 ribosomal protein L22 like 1(RPL22L1)

S100P S100 calcium binding protein P(S100P)

LOC100500766 no name found

RPL34A no name found

RPS17.S no name found

RPL31-PS12 no name found

RPS2E no name found

SULT5A1 no name found

RPL43A no name found

RPS-2 no name found

RPS51 no name found

RPS1101 no name found

RPL29.S no name found

RPL36.L no name found

RPS7A no name found

FAM189A2 no name found

RPS26L no name found

RPS-8 no name found

RPL35B no name found

C1QTNF6KCNJ11 no name found

RPS3A1 no name found


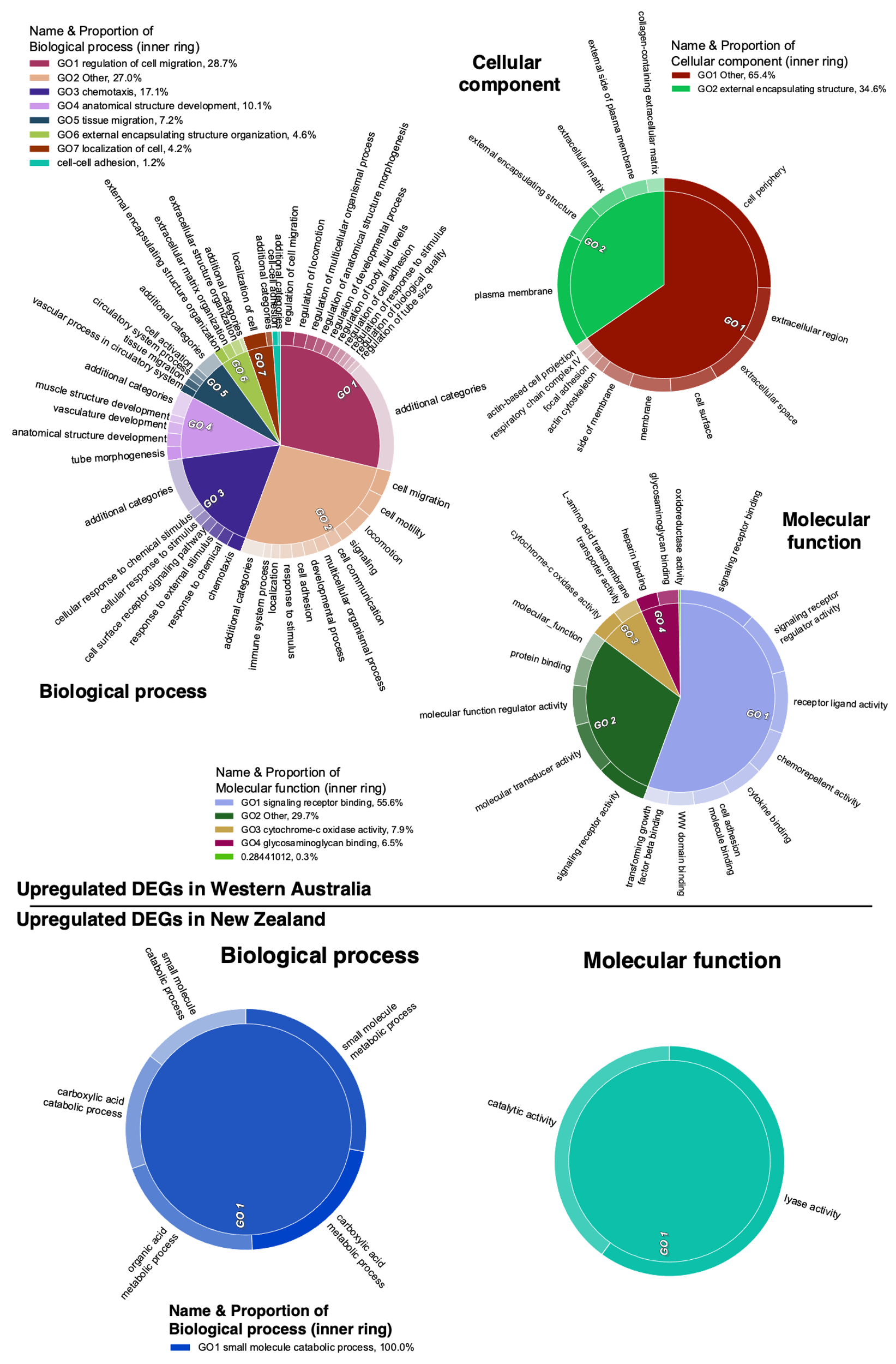


**Supplementary Fig. 8:** Gene Ontology (GOs) enrichment maps analysis among the significantly differentially regulated genes from liver samples isolated using Limma between toxin-resistant (Western Australian) and toxin-susceptible (New Zealand) brushtail possums (*Trichosurus vulpecula*). Terms are grouped by hierarchical clustering. Parent terms are identified in the legend along with their respective proportions, which are directly proportional to statistical significance. GO terms were first summarized based on a semantic similarity of 0.4 using REVIGO and visualized in CirGO. Circles correspond to one ontology group (BP: Biological process, MF: Molecular function and CC: Cellular component) and are separated between the GOs associated with the upregulated DEGs in Western Australia and in New Zealand.

**
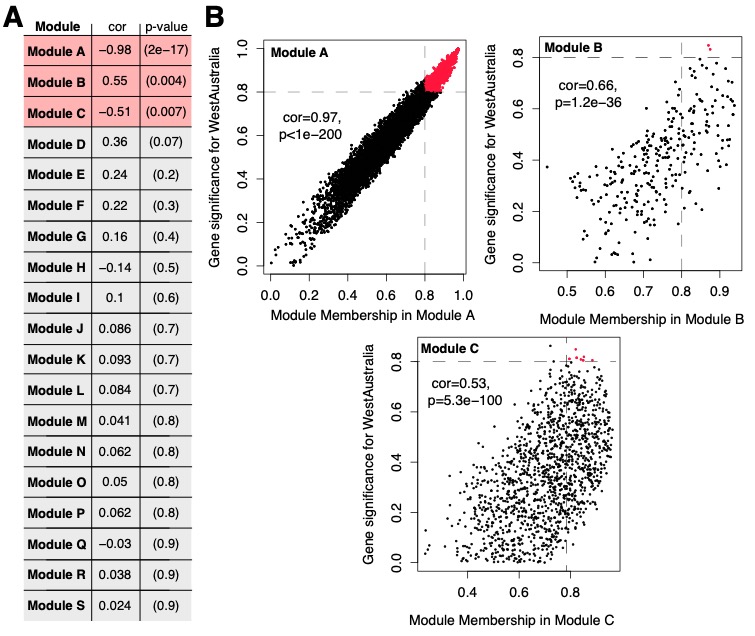
**

**Supplementary Fig. 9:** A) Association of each gene module with Western Australian (toxin resistant) brushtail possums. The first value of each cell corresponds to the correlation score and the value in brackets corresponds to the p-value of the correlation. B) Plots of Module Membership and Gene significance for maturity for each of the three significant modules. The associated correlation score and the p-value are indicated on each plot, and MM and GS thresholds used to select significant genes are shown with red lines. Significant genes for each module are coloured in red.

**
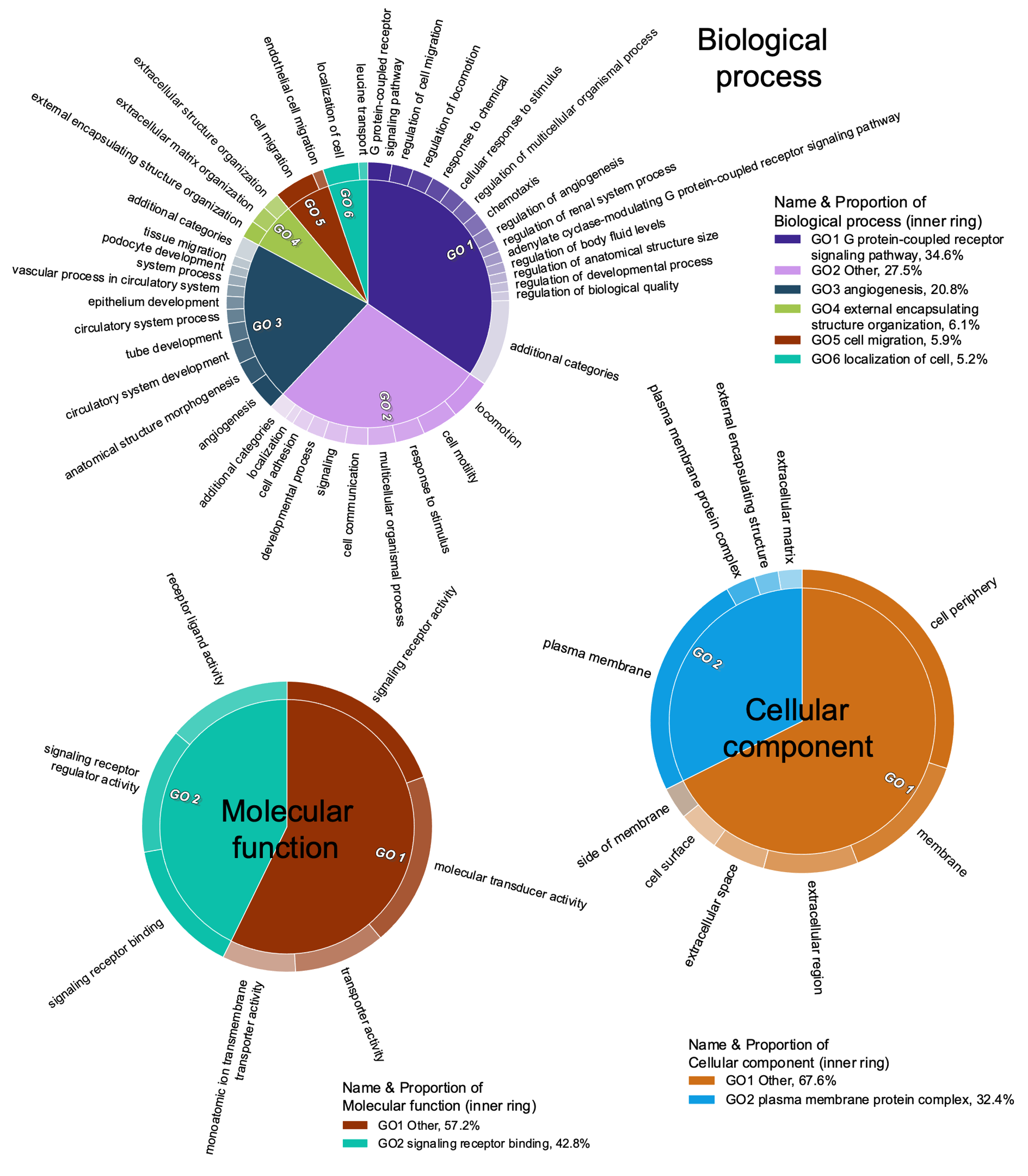
 Supplementary Fig. 10:** Gene Ontology (GOs) enrichment analysis among the significantly differentially regulated genes isolated using WGCNA between western Australian and New Zealand brushtail possum (*Trichosurus vulpecula*) liver. Terms are grouped by hierarchical clustering. Parent terms are identified in the legend and their respective proportions, directly proportional to statistical significance. GO terms were first summarized based on a semantic similarity of 0.4 using REVIGO and visualized in CirGO. Circles correspond to one ontology group (BP: Biological process, MF: Molecular function and CC: Cellular component) and are separated between the GOs associated with the upregulated DEGs in western Australian and in New Zealand.

**
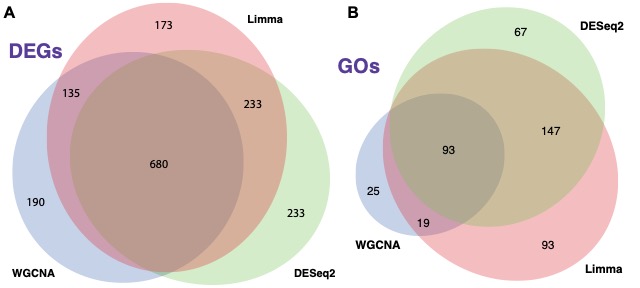
 Supplementary Fig. 11:** A: Visualization of the number of common differentially expressed genes (DEGs) using proportional Venn plots comparing the different packages used to identify gene expression differences in liver of brushtail possum. B: Visualization using proportional Venn plots of the number of enriched GO terms common to the three analyses.

**Supplementary Fig. 12:** Normalized counts of the ACO1 genes between western Australian (resistant to 1080-toxin; black dots) and New Zealand (susceptible to 1080-toxin; red dots) brushtail possums (*Trichosurus vulpecula*) liver RNASeq samples.
